# P38α/JNK signaling restrains erythropoiesis by suppressing Ezh2-mediated epigenetic silencing of Bim

**DOI:** 10.1038/s41467-018-05955-2

**Published:** 2018-08-29

**Authors:** Ping Hu, Angel R. Nebreda, Helmut Hanenberg, Garrett H. Kinnebrew, Mircea Ivan, Mervin C. Yoder, Marie-Dominique Filippi, Hal E. Broxmeyer, Reuben Kapur

**Affiliations:** 1 Department of Pediatrics, Herman B Wells Center for Pediatric Research, Indiana University School of Medicine, Indianapolis, Indiana, 46202 USA; 2 Institute for Research in Biomedicine (IRB Barcelona). Barcelona Institute of Science and Technology, Barcelona, 08028 Spain; 3 Department of Pediatrics III, University Children’s Hospital Essen, University of Duisburg-Essen, 45122 Essen, Germany; 4 Division of Hematology/Oncology, Department of Medicine, Indiana University School of Medicine, Indianapolis, Indiana, 46202 USA; 5 Division of Experimental Hematology and Cancer Biology, Cincinnati Children’s Research Foundation, University of Cincinnati College of Medicine, Cincinnati, OH 45229 USA; 6 Department of Microbiology/Immunology, Indiana University School of Medicine, Indianapolis, IN 46202 USA

## Abstract

While erythropoietin (EPO) constitutes the major treatment for anemia, a range of anemic disorders remain resistant to EPO treatment. The need for alternative therapeutic strategies requires the identification of mechanisms that physiologically restrain erythropoiesis. Here we show that P38α restrains erythropoiesis in mouse and human erythroblasts independently of EPO by integrating apoptotic signals during recovery from anemia. P38α deficiency promotes JNK activation through increased expression of Map3k4 via a negative feedback mechanism. JNK prevents Cdk1-mediated phosphorylation and subsequent degradation by Smurf2 of the epigenetic silencer Ezh2. Stabilized Ezh2 silences Bim expression and protects erythroblasts from apoptosis. Thus, we identify P38α/JNK signaling as a molecular brake modulating erythropoiesis through epigenetic silencing of Bim. We propose that inhibition of P38α, by enhancing erythropoiesis in an EPO-independent fashion, may provide an alternative strategy for the treatment of anemia.

## Abstract (web summary)

Erythropoietin (EPO) stimulates erythropoiesis and is commonly used to treat anemia. Here Hu et al. find that P38α/JNK signaling restrains erythropoiesis independently of EPO by regulating epigenetic silencing of the proapoptotic protein Bim, and thus identify putative targets for the treatment of anemic disorders resistant to EPO.

## Introduction

A remarkable feature of erythropoiesis is the coordination of proliferation, differentiation, and apoptosis of erythroid cells to precisely achieve erythropoietic homeostasis to avoid anemia and polycythemia^1^. Anemia is a common disease arising from various causes, including Myelodysplastic syndromes, thalassemia, cancer chemotherapy, chronic kidney disease, and hemorrhage^2^. The pro-erythropoietic factor erythropoietin (EPO) is often employed for anemia therapy. However, questions have been raised about the safety of EPO given its potential for tumor promotion in cancer-related anemia^3^. Moreover, many acute and chronic anemias, including hemolysis, sepsis, and genetic bone marrow failure diseases such as Diamond-Blackfan anemia are untreatable with EPO^4^. To overcome these hurdles, new molecular mechanisms need to be identified that physiologically restrain erythropoiesis by acting as molecular brakes to prevent over-active erythropoiesis caused by pro-erythropoietic signals. Inhibiting these restraining mechanisms could provide alternative approaches to treat anemia in an EPO-independent fashion.

P38 MAPK (Mitogen-activated protein kinase) is an important pathway involved in diverse biological processes. P38 modulates cell proliferation, controls cell survival and decides cell fate during differentiation. P38 pathway functions mainly by phosphorylating and activating important transcription factors in response to different stimuli, including ATF2, CREB, and MEF2^5^. There are four members within the P38 MAPK family, including P38α, P38β, P38γ, and P38δ. These members are encoded by different genes and have different tissue expression patterns. Among them, P38α is ubiquitously expressed. P38α modulates the function of different cell types^6^. There are two distinct developmental defects reported in global *P38α* knockout mice by two separate groups using different mouse strains. One displayed embryonic death with highly anemic appearance due to reduced EPO production and another showed even earlier embryonic lethality due to placental developmental defects^7,8^. In a *P38α* conditional mice model in which Cre recombinase was expressed in the whole-mouse embryo but not in the placenta by crossing to *MORE-Cre* mice, no anemia or EPO defects were observed^9^. However, the intrinsic and cell autonomous role of P38α in adult steady-state or stressful erythropoiesis has not been established. Loss of P38α causes activation of JNK in the liver^9^. P38 inhibitors are in clinical trials and have the potential for the treatment of human disease. Therefore, it is important to understand the downstream targets and functional outcomes induced by P38α deficiency.

Using primary human erythroblasts derived from human CD34^+^ hematopoietic stem and progenitor cells (HSPCs) and P38α conditional knockout mice, we find that P38α acts as a molecular brake during anemia recovery through integrating apoptotic signals and by shortening the lifespan of erythroblasts to prevent potential over-active erythropoiesis caused by pro-erythropoietic signaling. Loss of P38α in erythroblasts activates JNK through augmented Map3k4 via a negative feedback circuit revealed by gene expression profiling. Functionally, JNK serves as a pro-survival signal independent of EPO by compromising Bim expression via stabilizing the epigenetic silencer Ezh2 in erythroblasts. JNK-controlled Cdk1 activity modulates full interaction of Ezh2 to the E3 ligase Smurf2 through multiple threonine phosphorylation sites within Ezh2. Our findings identify a key signaling cascade involving P38α/JNK/Cdk1/smurf2/Ezh2/Bim in fine tuning stress erythropoiesis.

## Results

### Self-restraint role of P38 in stress erythropoiesis

**Fig. 1:**
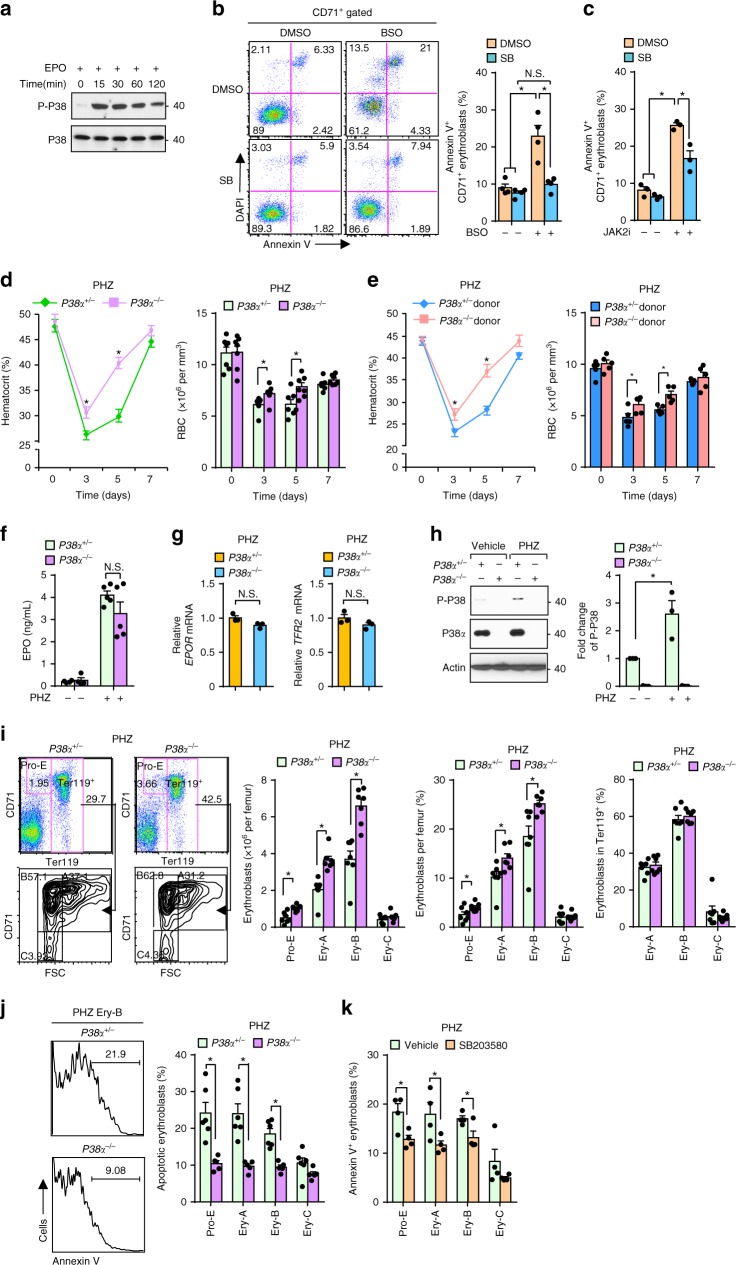
Self-restraint role of P38 in stress erythropoiesis. **a** Phosphorylation of P38 in starved human CD34^+^ HSPCs-derived erythroblasts stimulated with EPO (2 U ml^−1^) at indicated time points. **b**, **c** Apoptosis in human CD34^+^ HSPCs-derived erythroblasts induced by BSO (200 nM) (*n* = 4) (**b**) or Jak2 inhibitor II (40 µM) (*n* = 3) (**c**) with DMSO or SB203580 (10 µM) quantified by flow cytometry using Annexin V and DAPI staining. **d** PHZ-induced anemia and recovery in *P38α*^*+/*−^ and *P38α*^−*/*−^ mice. Analysis of Hematocrits (left) and RBC counts (right) were shown (*n* = 7). **e** Lethally irradiated C57BL/6-CD45.1 recipient mice were transplanted with bone marrow cells from *P38α*^*+/*−^ and *P38α*^−*/*−^ mice. Chimeric mice were challenged with PHZ and HCT and RBC counts were assessed (*n* = 5). **f**, **g** EPO serum concentration was measured by ELISA in *P38α*^*+/*−^ and *P38α*^−*/*−^ mice treated with vehicle (*n* = 4) or PHZ (*n* = 5) (**f**) and mRNA levels of EPOR and TFR2 (**g**) in PHZ-challenged *P38α*^*+/*−^ and *P38α*^−*/*−^ mice. **h**–**j** During recovery (day 4) from PHZ-induced anemia in *P38α*^*+/*−^ and *P38α*^−*/*−^ mice, western blot (left) and densitometry analysis (right) of phospho-P38 in BM erythroblasts. Data are shown as mean ± s.e.m from three separate experiments (**h**). Representative flow cytometry plot (left), numbers and percentages of BM erythroblast subsets (middle), and frequencies of Ery-A, Ery-B, and Ery-C within Ter119^+^ population (right) (*n* = 7) (**i**). Representative flow cytometry profile (left) and quantification (right) of BM erythroblast subsets undergoing apoptosis in *P38α*^*+/*−^ (*n* = 6) and *P38α*^−*/*−^ mice (*n* = 5) (**j**). **k** Mice were injected intraperitoneally with either SB203580 (15 mg per kg body weight) or vehicle every other day before and during PHZ challenge and BM apoptotic erythroblast subsets were measured as in **j** (*n* = 4). Blots are representative of three independent experiments. Data are shown as mean ± s.e.m. *P < 0.05 (two-tailed unpaired Student’s *t*-test)

Human CD34^+^ HSPCs were induced to undergo erythroid differentiation after stimulation with pro-erythropoietic factors erythropoietin (EPO) and stem cell factor (SCF), this provides a valuable tool to study human erythropoiesis in vitro^10,11^. The developmental characteristics of CD34^+^ HSPCs-derived erythroblasts were evaluated by examining the expression of cell surface markers CD71 and CD235a and by cell morphology (Supplementary Fig. 1a). We detected a more enduring and sustained activation of P38 in those cells stimulated by EPO than by SCF, suggesting a physiologic role for P38 in erythropoiesis (Fig. 1a and Supplementary Fig. 1b). To address the role of P38 in regulating erythroblasts, we found that SB203580, a specific inhibitor of P38, which inhibited the phosphorylation of ATF2 (a well-known P38 target), did not alter erythroid differentiation of HSPCs (Supplementary Fig. 1c, d). Cell cycle distribution was similar between control and P38-inhibited human erythroblasts using ki67 staining^12^ (Supplementary Fig. 1e). Given the association between elevated P38 activity and stress-induced apoptosis, we assessed whether suppression of P38 activity protected erythroblasts from oxidative stress or chemotherapy-triggered cell death, which is often observed in anemic patients. Buthionine sulfoximine (BSO) causes oxidative stress by depleting intracellular glutathione. Inhibition of P38 markedly elevated resistance of human erythroblasts to BSO-induced cell death without disturbing the cell cycle (Fig. 1b and Supplementary Fig. 1f). Inhibition of P38 also notably reduced cisplatin-induced apoptosis of human erythroblasts with no profound effects on cell cycle status (Supplementary Fig. 1g, h). We predicted that pro-survival signaling downstream of EPO and SCF would compromise pro-apoptosis P38 signaling in human erythroblasts under normal conditions. As expected, inhibition of Jak2, which inhibited the phosphorylation of stat5 (a well-known Jak2 target), uncovered the pro-apoptotic function of P38 (Fig. 1c and Supplementary Fig. 1i, j). Therefore, our finding that pro-apoptosis P38 pathway is activated by pro-erythropoietic signals such as EPO and SCF indicate that P38 potentially performs an intrinsic restraining function to limit over-active erythropoiesis by integrating apoptotic signals.

To further explore the role of P38 in erythropoiesis in vivo, we generated a conditional knockout mouse model in which *P38α* was deleted in hematopoietic cells by crossing *P38α*^*flox/flox*^ mice with *Mx-Cre* mice^6,13^. *P38α* locus was excised and P38α protein was completely abolished in sorted erythroblasts from *P38α*^−*/−*^ mice after poly IC injections compared to littermate controls (*P38α*^*+/−*^ mice) (Supplementary Fig. 2a). In steady-state erythropoiesis, loss of P38α showed normal peripheral blood erythroid parameters, including hematocrits (HCT) and RBC counts (Fig. 1d, 0 time point). Differentiation of mouse erythroblasts can be monitored by assessing the expression different cell surface proteins^14,15^. We next examined erythroblast differentiation characteristics by monitoring cell surface expression of CD71 and Ter119 combined with forward scatter (FSC) using flow cytometry^14^. A modest increase in pro-erythroblasts (Pro-E) associated with reduced apoptosis was observed in *P38α*^*−/−*^ bone marrow (BM) cells relative to controls; whereas, the frequency, viability and proliferation of basophilic erythroblasts (Ery-A), late basophilic and polychromatic erythroblasts (Ery-B) and orthochromatic erythroblasts (Ery-C) was comparable between *P38α*^*+/−*^ and *P38α*^−*/−*^ mice (Supplementary Fig. 2b–d). No obvious difference was observed in the composition of splenic erythroblast subsets (Supplementary Fig. 2e–g). These results demonstrate that P38α may modulate survival of early erythroblasts in BM under steady-state conditions, but loss of P38α is not sufficient to perturb erythropoietic homeostasis.

To investigate whether P38α modulates the response to anemia, we subjected *P38α*^*+/*−^ and *P38α*^−*/*−^ mice to phenylhydrazine (PHZ) treatment to induce hemolytic anemia. In response to PHZ challenge, *P38α*^−*/*−^ mice showed accelerated recovery of HCTs and RBC counts in peripheral blood compared to *P38α*^*+/*−^ mice (Fig. 1d). To further evaluate cell autonomous role of P38α in stress erythropoiesis, we transplanted BM cells from *P38α*^*+/*−^ and *P38α*^−*/*−^ mice into lethally irradiated recipient *C57BL/6-CD45.1* mice. After donor chimerism stabilized (~90%), transplanted mice were subjected to PHZ challenge. An accelerated erythropoietic recovery from anemia was observed in recipient mice transplanted with *P38α*^−*/*−^ BMs, suggesting the function of P38α in stress erythropoiesis is cell intrinsic (Supplementary Fig 3a and Fig. 1e).

EPO concentration in the serum and expression of *EPOR* and *TFR2* in erythroblasts was comparable between *P38α*^*+/*−^ and *P38α*^−*/*−^ mice after PHZ challenge, (Fig. 1f, g). During recovery from anemia, a significant increase in phosphorylated P38 was unexpectedly observed in erythroblasts (Fig. 1h) and *P38α*^−*/*−^ mice exhibited increase in the number of Pro-E, Ery-A and Ery-B cells in the BM and in the spleen compared to controls (Fig. 1i and Supplementary Fig. 3b). Although the frequency of Pro-E, Ery-A, and Ery-B was greater in *P38α*^−*/*−^ BM compared to *P38α*^*+/*−^ BM, the frequency of Ery-A, Ery-B, and Ery-C within the Ter119^+^ population was comparable between *P38α*^*+/*−^ and *P38α*^−*/*−^ mice, suggesting P38α may not be involved in modulating the differentiation of these subsets during stress erythropoiesis (Fig. 1i). PHZ-challenged mice showed enhanced cell cycle progression in erythroblasts in both *P38α*^*+/*−^ and *P38α*^−*/*−^ mice, demonstrating similar compensatory proliferation in erythroblasts during anemia recovery (Supplementary Fig. 3c, d). However, deletion of P38α led to reduced apoptosis in Pro-E, Ery-A, and Ery-B subsets both in the BM and in the spleen during recovery from anemia (Fig. 1j and Supplementary Fig. 3e). Administration of SB203580 also prevents apoptosis of BM erythroblasts without affecting cell cycle distribution in PHZ-challenged wild-type mice (Fig. 1k and Supplementary Fig. 3f). These results suggest that P38 integrates apoptotic cues in erythroblasts during the recovery from anemia to potentially limit over-active erythropoiesis.

Next, we challenged the mice with 5-fluorouracil (5-FU) (Supplementary Fig. 4a). During the recovery of this central anemia model, which induces anemia by depletion of proliferating progenitor cells, an enhanced P38 activity was observed (Supplementary Fig. 4b) and *P38α*^−*/*−^ mice exhibited accelerated erythropoietic recovery (Supplementary Fig. 4c), associated with increase in the numbers of Pro-E, Ery-A, and Ery-B and reduced apoptosis relative to *P38α*^*+/*−^ mice (Supplementary Fig. 4d, e). Thus, activation of P38 represents a physiologic molecular brake during the recovery phase of anemia by regulating erythroblast apoptosis; suppression of P38 activity relieves this restriction and promotes stress erythropoiesis.

### Loss-of-P38α drives JNK activation via augmented Map3k4

To understand how P38α deficiency exerts a pro-survival function in erythroblasts, we performed gene expression profiling in *P38α*^*+/*−^ versus *P38α*^−*/*−^ BM erythroblasts during the recovery of anemia in response to PHZ challenge. A total of 1086 genes were identified to be differentially expressed (DE) between *P38α*^−*/*−^ and *P38α*^*+/*−^ erythroblasts (*q*-value ≤0.05; fold change ≥2). Ingenuity pathway analysis (IPA) revealed “cell death and survival” as the most enriched molecular and cellular functional category from DE genes (*q*-value ≤0.05; fold change ≥1.5) (Supplementary Fig. 5a). Gene set enrichment analysis (GSEA) identified enriched Molecular Signature database gene sets in *P38α*^−*/*−^ erythroblasts, including suppression in the “inflammatory response pathway” (Supplementary Fig. 5b). Consistently, we detected reduced expression of TNF-α in *P38α*^−*/*−^ erythroblasts compared with *P38α*^*+/*−^ erythroblasts (Supplementary Fig. 5c).

**Fig. 2:**
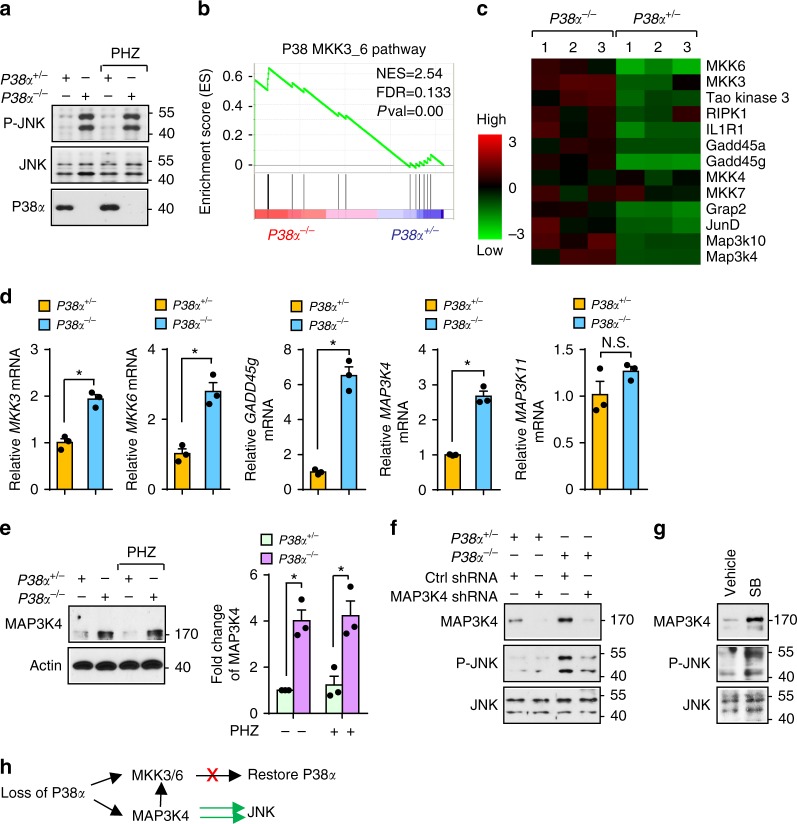
Upregulated Map3k4 due to P38α deficiency mediates JNK activation in erythroblasts. **a** In sorted erythroblasts from *P38α*^*+/*−^ and *P38α*^−*/*−^ mice, phosphorylated JNK (46 and 54 kDa) was detected by immunoblotting. **b** Gene set enrichment analysis (GSEA) of the P38 pathway based on gene expression profiling of sorted CD71^high^Ter119^+^ erythroblasts from *P38α*^*+/*−^ and *P38α*^−*/*−^ mice during recovery from PHZ challenge. NES normalized enrichment score; FDR false discovery rate *q*-value. **c** Heat map of P38α-related genes. **d** mRNA levels of key P38 and JNK regulatory genes in sorted *P38α*^*+/*−^ and *P38α*^−*/*−^ CD71^high^Ter119^+^ erythroblasts by q-PCR. **e** Western blot (left) and densitometry analysis (right) of Map3k4 protein level in *P38α*^*+/*−^ and *P38α*^−*/*−^ erythroblasts. Data are shown as mean ± s.e.m from three separate experiments. **f** Sorted GFP^+^ *P38α*^*+/*−^ and *P38α*^−*/*−^ cells transduced with control or Map3k4-specific shRNA. Expression of phosphorylated JNK and Map3k4 as assessed by immunoblotting. **g** Expression of phosphorylated JNK and Map3k4 in human erythroblasts cultured with DMSO or SB203580. **h** Schematic diagram demonstrating mechanism by which loss of P38α rewires increased Map3k4 to exclusively activate JNK. Blots are representative of three independent experiments. Data are shown as mean ± s.e.m. *P < 0.05 (two-tailed unpaired Student’s *t*-test)

Gene expression analysis revealed elevated expression of JunD, a downstream target of JNK, in *P38α*^−*/*−^ erythroblasts. We wondered whether JNK is activated in erythroblasts in response to loss of P38α as reported in liver^9^. *P38α*^−*/*−^ erythroblasts showed similar JNK activation under steady-state and stress conditions compared to *P38α*^*+/*−^ erythroblasts (Fig. 2a). Unexpectedly, GSEA revealed upregulation of the “P38 MKK3/6 pathway” in *P38α*^−*/*−^ erythroblasts, implying that a negative feedback circuit may occur in *P38α*^−*/*−^ erythroblasts in order to restore P38α signaling (Fig. 2b, c). q-PCR confirmed enhanced expression of MKK6, MKK3, and GADD45g (Fig. 2d), two direct upstream kinases and a protein responsible for promoting P38 activation^5,16^, but not MKK4 and MKK7, the direct upstream kinases of JNK, in *P38α*^−*/*−^ erythroblasts (Supplementary Fig. 5d). Enhanced expression of other P38 upstream kinases like Tao kinase 3^17^ and RIPK1^18^ were also noted in our gene expression profile (Fig. 2c). Remarkably, gene expression profiling also revealed upregulation in the expression of Map3k4 and Map3k10, which are upstream kinases of MKKs and mediate activation of P38^19,20^, in *P38α*^−*/*−^ erythroblasts (Fig. 2c). Increased Map3k4 expression was confirmed in *P38α*^−*/*−^ erythroblasts, whereas expression of Map3k11 was unaltered, with Map3k10 mRNA level too low to detect (Fig. 2d, e). These findings highlight the importance of P38α in stress erythropoiesis and demonstrate that negative feedback-induced augmentation of the upstream kinase of P38α is not limited to MKKs but acts upstream to the level of Mapkkks, in an attempt to restore P38α signaling.

An important feature of Map3k4 is to activate both P38 and JNK^20^. We reasoned that enhanced Map3k4 expression in *P38α*^−*/*−^ cells exclusively activates JNK due to lack of competition from P38α. Interestingly, elevated GADD45g can collaborate with Map3k4^16,21^. As expected, silencing Map3k4 by delivering a retrovirus containing shRNA against Map3k4 into *P38α*^−*/*−^ and *P38α*^*+/*−^ cells effectively ablated P38α deficiency-induced activation of JNK (Supplementary Fig. 5e and Fig. 2f). In human erythroblasts, inhibition of P38 by SB203580 activated JNK and this was associated with elevated expression of Map3k4 (Fig. 2g). This reveals a conserved mechanism in mice and humans which involves rewiring of JNK activation in the absence of P38α (Fig. 2h).

### JNK signaling protects erythroblasts independent of EPO

**Fig. 3:**
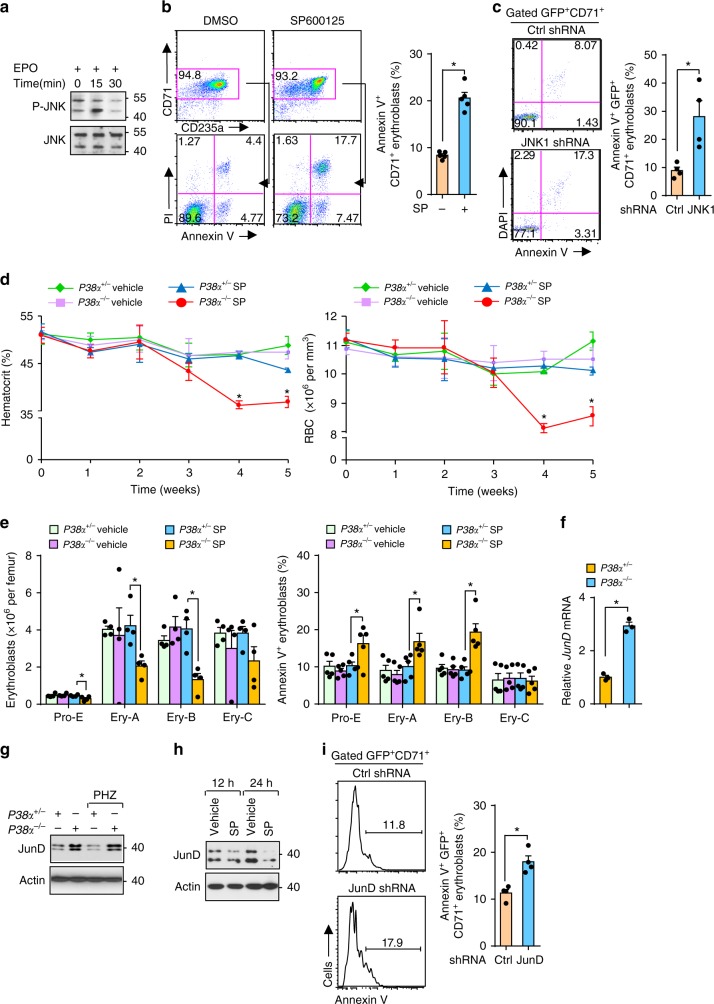
JNK promotes survival of erythroblasts independent of EPO. **a** Phosphorylation of JNK in starved human erythroblasts stimulated with EPO (2 U ml^−1^) at indicated time points. **b** Representative flow cytometry profile (left) and quantification (right) of apoptotic human erythroblasts induced by SP600125 (20 µM) for 36 h (*n* = 5). **c** Representative flow cytometry plots (left) and quantification (right) of apoptotic human erythroblasts by JNK1-specific shRNA (*n* = 4). **d** HCT and RBC counts on day 0 (*n* = 8) and on indicated time points (*n* = 5) in *P38α*^*+/*−^ and *P38α*^−*/*−^ mice treated with vehicle or SP600125. **e** Number (*n* = 4) and apoptosis (*n* = 5) of erythroblast subsets at fourth week in *P38α*^*+/*−^ and *P38α*^−*/*−^ mice exposed to vehicle or SP600125. **f**, **g** mRNA (**f**) and protein (**g**) expression of JunD from sorted *P38α*^*+/*−^ and *P38α*^−*/*−^ erythroblasts during recovery (day 4) from PHZ-induced anemia. **h** JunD protein levels in SP600125 (20 µM) treated human erythroblasts by immunoblotting. **i** Representative flow cytometry profile (left) and quantification (right) of apoptotic human erythroblasts induced by JunD-specific shRNA (*n* = 4). Blots are representative of three independent experiments. Data are shown as mean ± s.e.m. *P < 0.05 (two-tailed unpaired Student’s *t*-test)

Since loss of Map3k4 leads to apoptosis in certain tissues^20^ and JNK activity supports the survival of acute myeloid leukemia cells^22^, we questioned if JNK plays a role in modulating erythroblast function. We first examined whether JNK can be activated by EPO in human erythroblasts. Unlike P38, a relatively high basal JNK activity was observed in human erythroblasts, and EPO stimulation resulted in moderate and transient JNK activation, suggesting a limited role for EPO in maintaining JNK activity (Fig. 3a). Surprisingly, SP600125, a JNK kinase inhibitor, was sufficient to trigger apoptosis in human erythroblasts (Fig. 3b). We also observed apoptosis in human erythroleukemic TF1 cells in the presence of a JNK inhibitor (Supplementary Fig. 5f). Since TF1 cells do not rely on EPO for growth, the requirement for JNK activity for their survival suggests that JNK acts as a safeguard in the erythroid lineage independent of EPO. Consistent with the pharmacological results, silencing JNK1 by introducing a shRNA against JNK1 into human erythroblasts induced cell death (Supplementary Fig. 5g–i and Fig. 3c). Although both small molecule inhibitors and shRNA knockdown have off-target effects, combined results from mouse models, shRNA knockdown, and small molecule inhibitors might mitigate concerns associated with possible off-target effects to a large extent.

Since activated JNK contributed to the enhanced survival seen in *P38α*^−*/*−^ mouse erythroblasts similar to human erythroblasts, we predicted that *P38α*^−*/*−^ erythroblasts would be more vulnerable to JNK inhibition. Indeed, SP600125 administration in *P38α*^−*/*−^ mice resulted in an early and more severe decline in HCTs and RBCs, and reduction in the numbers of BM Pro-E, Ery-A, and Ery-B subsets, which was accompanied by enhanced apoptosis (Supplementary Fig. 5j and Fig. 3d, e). The fact that dependence on high JNK activity sensitizes *P38α*^−*/*−^ mice to JNK inhibition-induced anemia through increased apoptosis of erythroblasts further demonstrates that the JNK pathway acts as a pro-survival signal in erythroblasts.

JunD transduces pro-survival signals downstream of JNK^23^. Elevated expression of *JunD* mRNA and protein were observed in *P38α*^−*/*−^ erythroblasts with high JNK activity compared to P38α^+/−^ erythroblasts (Fig. 3f, g). In human erythroblasts, inhibition of JNK substantially ablated expression of JunD (Fig. 3h). Functionally, knockdown of JunD resulted in apoptosis of human erythroblasts more moderately than JNK1 silencing (Supplementary Fig. 5k and Fig. 3i), suggesting there might be other targets of JNK, in addition to JunD, involved in protecting erythroblasts.

### JNK protects erythroblasts by compromising Bim expression

**Fig. 4:**
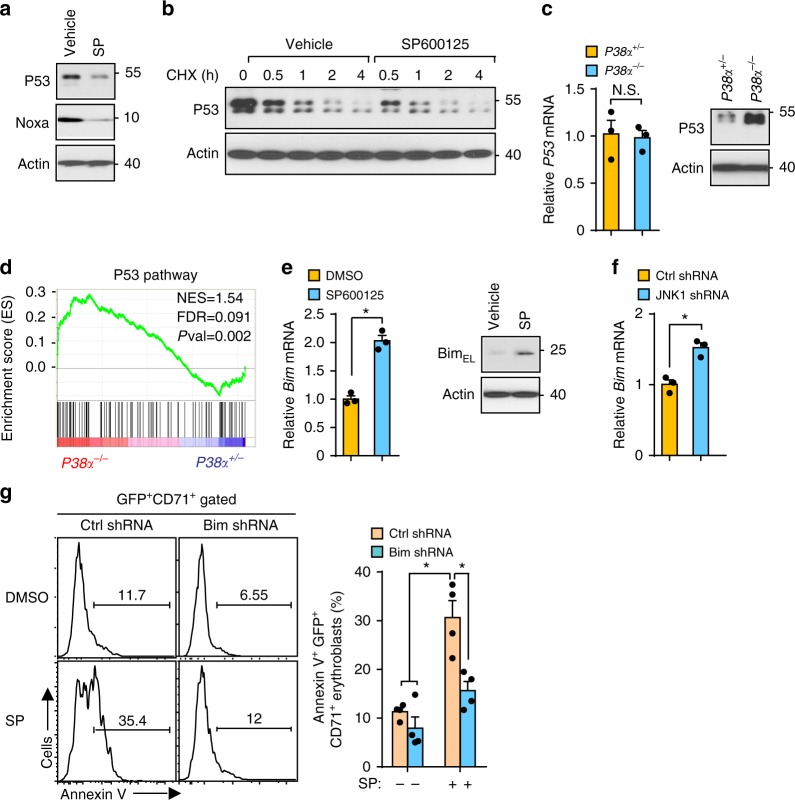
Bim but not P53 modulates erythroblast apoptosis downstream of JNK. **a** Human erythroblasts treated with DMSO or SP600125, P53 and Noxa expression measured by immunoblotting. **b** Time course of P53 protein expression in human erythroblasts treated with DMSO or SP600125 in the presence of cycloheximide (CHX). **c** P53 mRNA (left) and protein (right) level in *P38α*^*+/*−^ and *P38α*^−*/*−^ erythroblasts measured by q-PCR or western blot. **d** GSEA analysis of P53 pathway in *P38α*^*+/*−^ and *P38α*^−*/*−^ erythroblasts. **e** mRNA (left) and protein (right) level of Bim in human erythroblasts triggered by SP600125 at 12 h by q-PCR or immunoblotting. **f** Bim mRNA expression in human erythroblasts induced by JNK1 shRNA. **g** Representative flow plots (left) and Quantification (right) of SP600125-induced apoptosis of human erythroblasts transduced with control or Bim-specific shRNA (*n* = 4). Blots are representative of three independent experiments. Data are shown as mean ± s.e.m. *P < 0.05 (two-tailed unpaired Student’s *t*-test)

Since the tumor suppressor P53 is a master regulator of apoptosis in many cell types, including erythroblasts^24^, and mutations in the ribosomal protein genes cause erythroid cell death in patients with Diamond-Blackfan anemia via P53 activation^11^, we examined whether P53 is involved in JNK inhibition-induced apoptosis in human erythroblasts. We evaluated the impact of JNK inhibition on expression of P53, and surprisingly found that protein levels of P53 and Noxa, a well-known P53 target, were markedly reduced upon inhibition of JNK in human erythroblasts (Fig. 4a). Cycloheximide (CHX)-chase assay revealed that JNK inactivation triggers accelerated degradation of P53 compared to controls (Fig. 4b), suggesting that JNK activity is essential for maintaining stability of P53 in human erythroblasts. Moreover, *P38α*^−*/*−^ erythroblasts, with high JNK activity, exhibited increased expression of P53 protein, but not *P53* mRNA, compared to *P38α*^*+/*−^ erythroblasts (Fig. 4c). This is consistent with GSEA analysis that displayed enhanced enrichment of the P53 pathway in *P38α*^−*/*−^ erythroblasts (Fig. 4d). These data suggest that P38α is a physiological negative regulator of P53 via JNK and that P53 might not contribute to JNK inhibition-induced apoptosis in erythroblasts.

To address whether cell death receptor pathways which play a critical role in controlling erythroblast survival are downstream targets of JNK and responsible for mediating erythroblast cell death, we performed q-PCR analysis. As seen in Supplementary Fig. 6a, JNK inactivation did not significantly alter the expression of *Fas*, *DR3*, and *DR4* but decreased the expression of *FasL*. Bcl-2 family proteins play a central role in regulating erythroblast cell death^25,26^. Among the Bcl-2 family members, we found the expression of *Bim*, but not *bcl-Xl*, *Mcl-1*, and *Bik*, to be significantly elevated by SP600125 (Fig. 4e and Supplementary Fig. 6b). Knockdown of JNK1 by shRNA also activated Bim expression in these cells (Fig. 4f). Importantly, knockdown of Bim by lentivirus carrying Bim-specific shRNA enabled erythroblasts to resist JNK inactivation-induced cell death (Supplementary Fig. 6c and Fig. 4g). Thus, the JNK pathway supports the survival of erythroblasts by regulating the expression of Bim.

### Smurf2-mediated degradation of Ezh2 triggers Bim expression

Elevated Bim mRNA levels imply that JNK perhaps modulates Bim transcription. Therefore, we focused on transcription factors Foxo3a and LRF, both of which are involved in regulating Bim expression and erythropoiesis^25,27^. Phosphorylation of Foxo3a inhibits its function^28^. We found Foxo3a phosphorylation to be unaltered in response to SP600125. Expression of LRF protein remained unchanged by JNK inactivation (Supplementary Fig. 7a). These results suggest that both Foxo3a and LRF might not be involved in regulating JNK-mediated Bim expression. This prompted us to seek alternative targets.

**Fig. 5:**
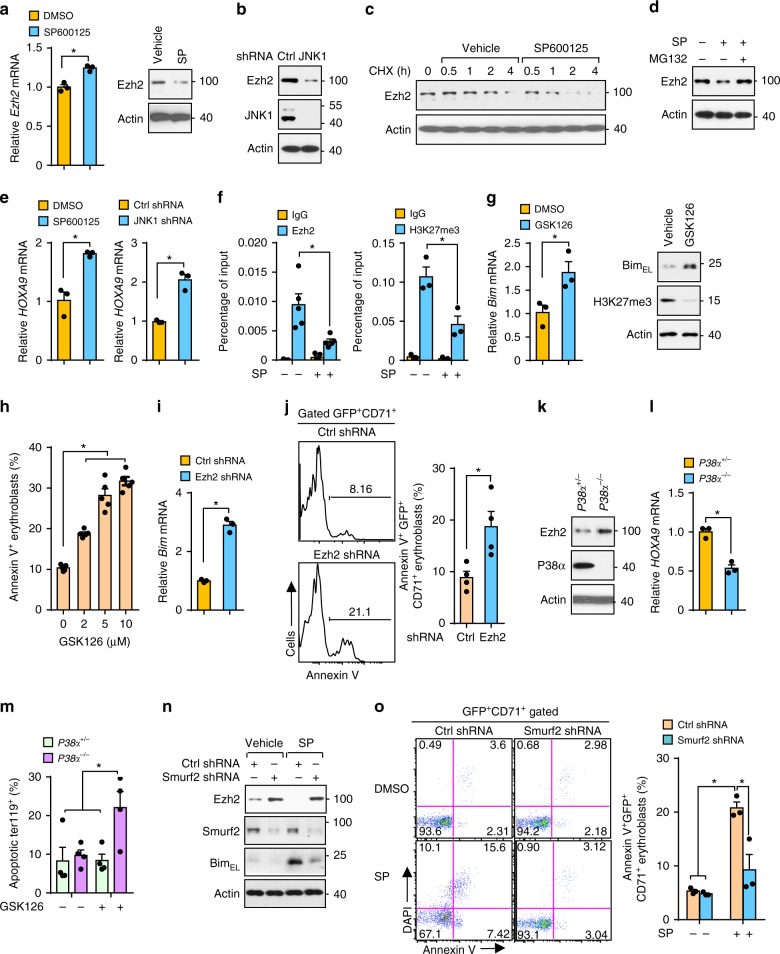
JNK inhibits Bim expression through suppressing Smurf2-mediated degradation of Ezh2. **a** mRNA (left) and protein (right) levels of Ezh2 in human erythroblasts triggered by SP600125 assessed by q-PCR or immunoblotting. **b** Ezh2 protein level induced by JNK1 silencing measured by immunoblotting. **c** Time course of Ezh2 levels in CHX-treated human erythroblasts with DMSO or SP600125. **d** SP600125-induced degradation of Ezh2 in the presence of DMSO or MG132 (10 µM) by immunoblotting. **e** mRNA expression of HOXA9 in human erythroblasts induced by SP600125 (left) or JNK1 shRNA (right) by q-PCR. **f** Quantitative chromatin immunoprecipitation analysis of occupancy of Ezh2 (left, *n* = 5) and H3K27me3 (right, *n* = 3) on Bim promoter in human erythroblasts treated with vehicle or SP600125. **g** mRNA (left) and protein (right) levels of Bim induced by GSK126 (5 µM) for 12 h. **h** Apoptosis of human erythroblasts trigged by GSK126 and measured by flow cytometry. **i** mRNA expression of Bim induced by Ezh2 silencing. **j** Representative flow cytometry profile (left) and quantification (right) of apoptotic human erythroblasts induced by Ezh2-specific shRNA (*n* = 4). **k** Ezh2 protein levels in *P38α*^*+/*−^ and *P38α*^−*/*−^ erythroblasts by immunoblotting. **l** mRNA levels of HOXA9 in *P38α*^*+/*−^ and *P38α*^−*/*−^ erythroblasts by q-PCR. **m** Apoptosis in *P38α*^*+/*−^ and *P38α*^−*/*−^ erythroblasts subjected to GSK126 (5 µM) (*n* = 4). **n** Protein levels of Ezh2, Smurf2, and Bim in human erythroblasts transduced with control or smurf2-specific shRNA then treated with SP600125. **o** Representative flow cytometry profile (left) and quantification (right) of SP600125-induced apoptosis of human erythroblasts transduced with control or smurf2-specific shRNA (*n* = 3). Blots are representative of two independent experiments. Data are shown as mean ± s.e.m. *P < 0.05 (two-tailed unpaired Student’s *t*-test)

Ezh2, a component of the polycomb repressive complex 2 (PRC2), has been reported to negatively regulate Bim expression^29,30^. Ezh2 regulates erythropoiesis^31,32^. We wondered whether Ezh2 is a downstream target of JNK in regulating Bim expression. In human erythroblasts, *Ezh2* mRNA levels were modestly elevated, whereas Ezh2 protein was markedly reduced upon inhibiting the activity of JNK (Fig. 5a). Silencing JNK1 also significantly attenuated Ezh2 expression (Fig. 5b). Cycloheximide chase assay performed in human erythroblasts revealed that inactivation of JNK notably accelerated degradation of Ezh2 protein, suggesting that JNK might control Ezh2 protein stability (Fig. 5c). Importantly, degradation of Ezh2 by JNK inactivation could be blocked by proteasome inhibitor MG132, suggesting the involvement of the ubiquitin-proteasome pathway in Ezh2 regulation (Fig. 5d). Consistently, elevated *HOXA9* mRNA level, a well-known target of PRC2, was observed in human erythroblasts when JNK activity was compromised by SP600125 or by JNK1 silencing (Fig. 5e). In agreement with these results, we found reduced binding of Ezh2 and H3K27 trimethylation on the *Bim* promoter, as measured by a quantitative chromatin immunoprecipitation assay (Fig. 5f).

We then tested the role of Ezh2 in modulating erythroblast survival. In human erythroblasts, GSK126, an Ezh2 inhibitor, induced Bim expression while compromising global H3K27me3 and initiated cell death in a concentration dependent manner (Fig. 5g, h and Supplementary Fig. 7b). Similar results were observed when Ezh2 was silenced by shRNA (Supplementary Fig. 7c and Fig. 5i, j). Ezh2 activity was also required for the survival of TF1 cells (Supplementary Fig. 7d). These results indicate that Ezh2 is required for protecting human erythroblasts by inducing epigenetic silencing at Bim locus. In accordance with findings in human erythroblasts, *P38α*^−*/*−^ erythroblasts, which show increased JNK activation, also displayed elevated Ezh2 protein compared with controls (Fig. 5k). Consistently, reduced expression of HOXA9 was observed in *P38α*^−*/*−^ erythroblasts compared to *P38α*^*+/*−^ erythroblasts (Fig. 5l). Moreover, *P38α*^−*/*−^ erythroblasts showed greater sensitivity to Ezh2 inhibition-induced apoptosis than P38^+/−^ erythroblasts, suggesting dependence of *P38α*^−*/*−^ erythroblasts on higher level of Ezh2 (Fig. 5m). Together, our findings demonstrate that Ezh2 is a target of the P38α/JNK pathway and is required for protecting erythroblasts by silencing Bim expression. Thus, our results link Ezh2, a major epigenetic regulator of gene expression, to major MAPK signaling pathway to define a mechanism for maintenance of erythropoietic homeostasis.

Smurf2, a ubiquitin E3 ligase, can degrade Ezh2^33^. To investigate the role of smurf2 in JNK-mediated Ezh2 protein stability in human erythroblasts, we silenced smurf2 by delivering lentivirus containing shRNA against smurf2 into human erythroblasts. This treatment enhanced the expression of Ezh2 under steady-state and JNK inactivation conditions compared to control shRNA and compromised the expression of Bim upon JNK inactivation (Supplementary Fig. 7e and Fig. 5n). Importantly, erythroblasts lacking Smurf2 showed enhanced resistance to apoptosis in response to JNK inhibition (Fig. 5o). These results support a key signaling cascade involving JNK/Smurf2/Ezh2 in regulating survival of erythroblasts.

### Cdk1 phosphorylates Ezh2 to mediate its binding to Smurf2

**Fig. 6:**
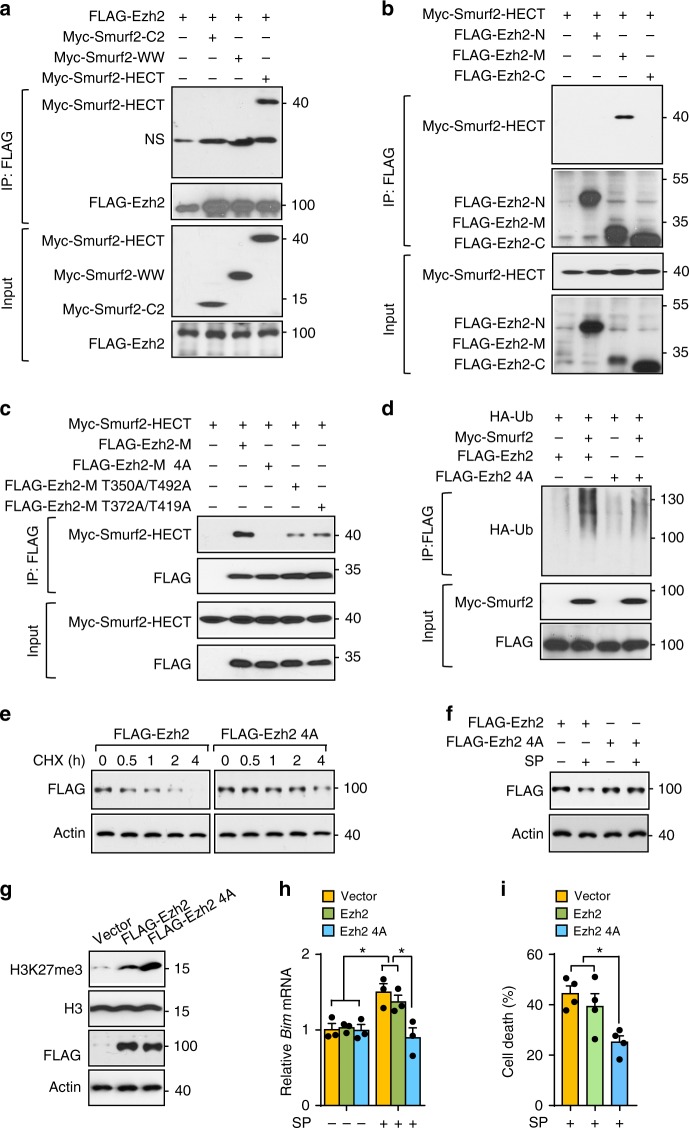
Multiple cdk1 threonine phosphorylation sites in Ezh2 modulate its full interaction with Smurf2. **a** HECT domain of Smurf2 interacting with Ezh2 by co-immunoprecipitation. **b** Binding of middle domain of Ezh2 (Ezh2-M) to Smurf2 by co-immunoprecipitation. **c** Ezh2-M wild type and mutants were co-transfected with HECT domain of Smurf2 and cell lysates were subjected to co-immunoprecipitation after pre-treatment with MG132 for 6 h. **d** Ubiquitination of wild-type Ezh2 and Ezh2 4A mutants in cells co-expressing HA-tagged ubiquitin with or without smurf2 in the presence of MG132. **e**, **f** Sorted GFP^+^ TF1 cells expressing Flag tagged wild type or 4A mutant Ezh2 were subjected to CHX treatment (**e**) or SP600125 (**f**) and Ezh2 protein abundance was measured by immunoblotting using anti-Flag antibody. **g**–**i** In sorted GFP^+^ TF1 cells expressing control or Flag tagged wild type or 4A mutant Ezh2, protein levels of H3K27me3 and total H3 levels measured by immunoblotting (**g**) mRNA expression of Bim by q-PCR (**h**) and cell death by MTT assay (*n* = 4) (**i**) after SP600125 treatment. Blots are representative of two independent experiments. Data are shown as mean ± s.e.m. *P < 0.05 (two-tailed unpaired Student’s *t*-test)

To further explore the mechanisms underlying Ezh2 regulation by Smurf2, we mapped the binding domain between Ezh2 and Smurf2. Smurf2 contains an N-terminal C2 domain and a C-terminal HECT domain which has E3 ligase activity with three WW domains in the middle^34^. Co-immunoprecipitation showed strong interaction between full-length Ezh2 and the HECT domain of Smurf2, but not the C2 or WW domains (Fig. 6a). Next, we mapped the region within Ezh2 that mediates interaction with the HECT domain of Smurf2. We designed a series of Ezh2 truncated constructs, including an N-terminal fragment (amino acid residues 1–348), referred to as Ezh2-N, middle region amino acid residues 330–530 which consist of multiple threonine phosphorylation sites referred to as Ezh2-M and a C-terminal domain containing amino acid residues 523–751 referred to as Ezh2-C. We observed strong binding of the HECT domain to the Ezh2-M fragment (Fig. 6b). These results demonstrate that the middle region of Ezh2 interacts with the C-terminal HECT domain of Smurf2.

There are four threonine phosphorylation sites within Ezh2-M, including Ter350, Ter372, Ter419, and Ter492. Three of them have been reported to be phosphorylated by Cdk1/2, and two of them (T350 and T492) regulate Ezh2 stability^35–38^. Although a previous report showed that T372 can be phosphorylated by P38^39^, motif analysis strongly predicts T372 as a CDK consensus site^40^. To this end, we generated one quadruple-point mutant with four threonine residues mutated to alanine (A) (referred to as Ezh2-M 4A), and one triple-point mutant with a T372 residue remaining intact (referred to as Ezh2-M 3A372T). Immunoprecipitation showed enhanced phosphorylation of the 3A372T mutant as revealed by a phosphorylated CDK substrate antibody (pT-P Ab) compared to a 4A mutant. Notably, Roscovitine, a CDK inhibitor, significantly ablated the phosphorylation of 3A372T mutant (Supplementary Fig. 8a). In support of this, co-expression of cdk1 and cyclin B significantly increased phosphorylation of the Ezh2-M 3A372T mutant, but not of the Ezh2-M 4A mutant (Supplementary Fig. 8b). These findings demonstrate that T372 can be phosphorylated by cdk1.

Since we noticed that Lysine-421^33^, which is critical for smurf2-mediated Ezh2 degradation, is surrounded by four threonine sites, we assessed if phosphorylation of these threonine sites mediates the interaction between Ezh2 and smurf2. Wild type (Ezh2-M) and a series of Ezh2 mutants including a quadruple threonine mutant (Ezh2-M 4A), two double threonine mutants (Ezh2-M T350A/T492A and Ezh2-M T372A/T419A) were co-expressed with the HECT domain of smurf2. Co-immunoprecipitation showed that the double threonine mutation in Ezh2-M significantly weakened the binding, compared to wild type, and the quadruple threonine mutation abolished the interaction completely (Fig. 6c). In support of these findings, in vivo ubiquitination assay showed Smurf2 caused dramatically reduced poly-ubiquitination of the 4A mutant of Ezh2 compared to wild-type Ezh2 (Fig. 6d). To further address the functional significance of the threonine phosphorylation sites in Ezh2, we overexpressed the full length wild type or 4A mutant of Ezh2 in TF1 cells. Compared to wild type, the 4A mutant of Ezh2 displayed enhanced stability in a CHX chase assay and showed resistance to degradation induced by JNK inactivation (Fig. 6e, f). It has been proposed that individual threonine sites may have distinct impact on Ezh2 activity^35,36^. Expression of the Ezh2 4A mutant significantly increased global H3K27 trimethylation compared with wild-type Ezh2 in sorted GFP^+^ TF1 cells (Fig. 6g). Importantly, expression of the Ezh2 4A mutant compromised the expression of *Bim* and protected TF1 cells from JNK inhibition-induced cell death compared to wild-type Ezh2 expressing cells (Fig. 6h, i). Our findings demonstrate that four threonine phosphorylation sites negatively regulate Ezh2 activity and are required for full interaction between Ezh2 and smurf2 for its degradation. Thus, regulation of Cdk1 activity is a key step in JNK-mediated modulation of Ezh2 function.

### Cdk1 modulates Ezh2 and survival of erythroblasts

**Fig. 7:**
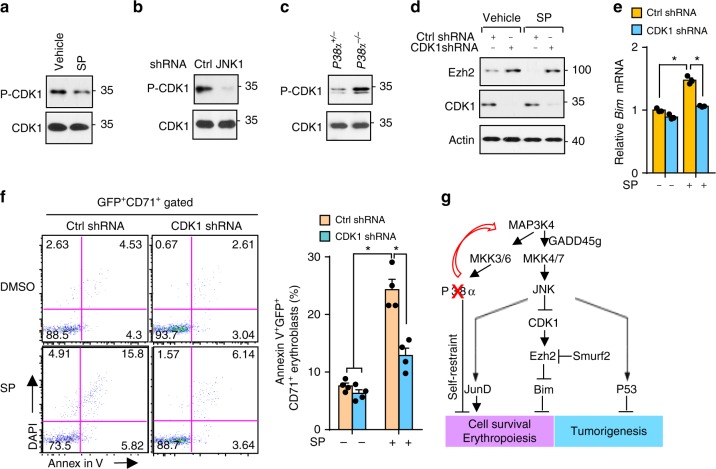
JNK-mediated Cdk1 inhibition stabilizes Ezh2 and supports erythroblasts. **a**, **b** Tyr-15 phosphorylation of Cdk1 in SP600125-treated (**a**) or in JNK1-silenced (**b**) human erythroblasts by immunoblotting. **c** Phosphorylation of Tyr-15 of Cdk1 in *P38α*^*+/*−^ and *P38α*^−*/*−^ erythroblasts. **d**–**f** Human erythroblasts transduced with control or cdk1-specific shRNA and treated with vehicle or SP600125. Protein levels of Ezh2 and Cdk1 by immunoblotting (**d**), mRNA expression of Bim by q-PCR (**e**), and representative flow cytometry profile (left) and quantification (right) of apoptotic human erythroblasts (**f**) (*n* = 4). **g** Schematic outlining a proposed mechanism mediating rewired JNK activation due to lack of P38 via Map3k4 and their downstream targets regulating erythropoiesis. Blots are representative of three independent experiments. Data are shown as mean ± s.e.m. *P < 0.05 (two-tailed unpaired Student’s *t*-test)

Next, we questioned how JNK controls the activity of Cdk1. De-phosphorylation of Tyr-15 is required for Cdk1 activation^41^. Suppression of JNK by SP600125 or JNK1 knockdown significantly reduced phosphorylation of Tyr-15 of Cdk1 in human erythroblasts (Fig. 7a, b). Conversely, elevated Cdk1 Tyr-15 phosphorylation was observed in *P38α*^−*/*−^ erythroblasts which bear enhanced JNK activity compared with *P38α*^*+/*−^ erythroblasts, implying that JNK modulates Cdk1 via Tyr-15 (Fig. 7c). To address the role of Cdk1 in the regulation of erythroblast survival, we silenced Cdk1 expression by a Cdk1-specific shRNA. This resulted in increased expression of Ezh2 under both basal and JNK inactivation conditions (Supplementary Fig. 8c and Fig. 7d). Importantly, lack of Cdk1 compromised the transcription of *Bim* and protected erythroblasts under JNK inactivation conditions (Fig. 7e, f). These findings demonstrate that Cdk1 functions downstream of JNK and bridges JNK activity to Ezh2 modulation. Our results thus establish a critical signaling cascade involving P38/JNK/Cdk1/Smurf2/Ezh2/Bim in controlling survival of erythroblasts.

## Discussion

Our work has uncovered a self-restraining function of P38α in stress erythropoiesis by integrating apoptotic signals in erythroblasts during the recovery from anemia. Therefore, P38α acts as a molecular brake to limit over-active erythropoiesis in response to stress. Relief of this molecular brake by inhibiting P38 enhances stress erythropoiesis and accelerates recovery from anemia, thus providing a potential therapeutic strategy for treating patients with anemia (Fig. 7g).

Both P38 and JNK belong to MAPK family. Our findings show that in human primary erythroblasts, EPO-induced activation of JNK only occurs briefly, whereas activation of P38 persists. The intensity, duration, and fluctuation of stimulation of signaling pathways are all critical in determining the eventual output to affect cell fate decisions^42^. Endurance of activated signaling pathways by EPO/EPOR is regulated by various mechanisms such as availability of adaptor proteins, endocytosis of EPO/EPOR^43^, expression of specific phosphatases, and cross-talk between multiple downstream pathways of EPO/EPOR^44^. Adding to the complexity, recent findings demonstrate that extracellular binding changes between EPO and EPOR due to EPO variant result in selectively altered downstream signaling responses, which reveals a mechanism in EPO/EPOR signaling modulation^45^.

Gene expression profiling did not reveal elevated expression of other members of the P38 kinase family (P38β/γ/δ) in *P38α*^−*/*−^ erythroblasts. Since P38β can also be activated by MKK6^46^, we have performed q-PCR experiments and found that expression of P38β in mouse erythroblasts was very low, which is in agreement with previous findings in human erythroblasts^47^, suggesting that P38β may not play an important role in regulation of erythropoiesis. The expression of P38γ was even lower than P38β in mice erythroblasts, although P38γ was found to express in human erythroblasts^47^. Hence, other members of P38 family may not compensate for the loss of P38α in mice erythroblasts, highlighting the importance of P38α in erythropoiesis.

We propose that the JNK/Ezh2 signaling serves as a pro-survival mechanism in erythroblasts independent of EPO. Data from us and others suggests that JNK activity is required for maintaining P53 protein. Loss-of-MKK7 inhibits JNK activity, accelerates P53 degradation, and promotes lung cancer development^48^. In contrast, in hematopoietic system-derived malignancies, like leukemia, which rarely bear mutations in P53 compared to solid tumors, JNK activity is required for leukemic cells to survive^22^. Our findings that inhibition of JNK causes apoptosis while promotes degradation of P53 in human erythroblasts, indicate that the consequence of JNK activation may depend on whether P53 plays an essential role in regulating apoptosis in specific cell types. Indeed, there is evidence to suggest that P53-independent regulation of apoptosis via LRF/Bim, Fas/FasL, and TRAIL exist in erythroid cells^14,25,49^. Our findings show that Ezh2 is a downstream target of P38α/JNK pathways, establishing a connection between critical P38α/JNK signals and PRC2, an essential epigenetic regulator. Our results reveal a signal transduction mechanism for sensing extracellular/intracellular changes to chromatin re-modeling, which enables erythroblasts to adapt to changing environment to maintain homeostasis.

Our findings demonstrate that Cdk1 functions downstream of JNK to regulate Ezh2 stability and its activity. Phosphorylation of four threonine sites in the middle domain of Ezh2 by Cdk1 mediates full interaction to bind to Smurf2 for its degradation. We propose a two-step mechanism by which JNK regulates Ezh2 via Cdk1 in erythroblasts. If JNK inhibition is short-term, activated Cdk1 phosphorylates and decreases Ezh2 activity to trigger target gene expression. However, if JNK inactivation persists, endured activated Cdk1 mediates phosphorylation and degradation of Ezh2 via Smurf2 for a longer duration compromising Ezh2 function.

P38 has been reported to positively regulate P53^50^. However, those results are mostly from cancer cell lines in which signal transduction usually has been dysregulated. In contrast, we observed enhanced P53 protein in *P38α*^−*/*−^ erythroblasts, suggesting that P38α physiologically negatively modulates P53. Recent studies show that P38 is a potential target for certain cancer therapies^51^. Our findings that P38 regulates P53 and Ezh2 through JNK further provides insight into the role of P38 in tumorigenesis (Fig. 7g). Given the fact that many anemias are caused by chemotherapy, we propose an alternative therapeutic strategy for improving the outlook in anemia patients through inhibition of P38. Inhibition of P38 not only promotes erythropoiesis by relief of self-restraint in erythroblasts but also potentially maintains a higher level of P53 in cancer cells which is doubly beneficial for cancer patients who bear wild-type P53.

## Methods

### Reagents and cell culture

P38 inhibitor SB203580, JNK inhibitor SP600125, Jak2 inhibitor II, and MG132 were purchased from EMD Bioscience. Cdk inhibitor Roscovitine was purchased from Cell Signaling technology. Cycloheximide was purchased from Sigma-Aldrich. Human Stem cell factor (SCF), thrombopoietin (TPO), and FLT3-ligand (FLT3L), human GM-CSF, murine IL-3 and SCF were purchased from Peprotech. EPO was manufactured by Amgen. Fetal bovine serum (FBS) was purchased from Hyclone. TF1 human erythroid cells (American Type Culture Collection (ATCC)) were cultured in RPMI 1640 medium supplemented with 10% FBS and GM-CSF (2 ng ml^−1^) and 1% penicillin/streptomycin. HEK293T (ATCC) and Hela cells (ATCC) were kept in DMEM supplemented with 10% FBS and 1% penicillin/streptomycin. All cell lines were authenticated by supplier. All cells were tested for mycoplasma contamination.

### Mice and animal studies

Mice carrying the floxed *P38α* alleles (*P38α*^*fl/fl*^) was kindly provided by Dr. Angel R. Nebreda^6,13^. *P38α*^*fl/fl*^ mice were mated among themselves and to *Mx1-Cre* mice (The Jackson Laboratory, Bar Harbor, ME) to create *Mx1-Cre*/*P38α*^*fl/+*^ (*P38α*^*+/*−^) mice and *Mx1-Cre*/*P38α*^*fl/fl*^ (*P38α*^−*/*−^) mice. Genotypes were confirmed by PCR of tail DNA. To induce the expression of Cre to delete floxed P38α alleles, 6- to 8-week-old male and female mice were injected intraperitoneally with 300 µg PolyIC every other day for three times and mice rested for 6 weeks prior to initiation of experiments, including gene analysis. All studies were performed in a *C57Bl/6* genetic background. Mice were randomized based on weight. The investigators were not blinded for group allocations. All mice were maintained in specific pathogen-free conditions, and experiments were approved by the Institutional Animal Care and Use Committee (IACUC) of the Indiana University School of medicine.

*P38α*^*+/*−^ and *P38α*^−*/*−^ mice were intraperitoneally injected with PHZ (100 mg per kg body weight) once to induce hemolytic anemia. For induction of central anemia, a single intraperitoneal injection of 5-fluorouracil (150 mg per kg body weight) was utilized. To inhibit JNK, *P38α*^*+/*−^ and *P38α*^−*/*−^ mice were injected daily intraperitoneally with SP600125 (40 mg per kg body weight). Peripheral blood was collected from tail vein at indicated times and hematological parameters were analyzed using a HEMAVET 950FS analyzer.

Administration of P38 inhibitor SB203580 to mice was performed^52^. In brief, Mice were injected intraperitoneally with SB203580 (15 mg per kg body weight) or vehicle every other day before (2 injections) and during PHZ challenge. Apoptosis and cell cycle of BM erythroblast subsets was measured by flow cytometry.

For the transplantation experiment, 1 × 10^6^ bone marrow cells from *P38α*^*+/*−^ and *P38α*^−*/*−^ mice (CD45.2^+^) were isolated and injected into lethally irradiated *C57BL/6-CD45.1* recipient mice (CD45.1^+^CD45.2^+^) through tail vein. The donor chimerism were monitored by flow cytometry analysis of peripheral blood samples with anti-mouse CD45.1-PE (553776, BD Bioscience) and CD45.2-FITC antibodies (553772, BD Bioscience) at a 1:50 dilution. After donor chimerism was stable over 90%, chimeric mice were subject to PHZ challenge.

### Lentiviral transduction and erythroid differentiation

Purified human cord blood CD34^+^ HSPCs were purchased from the Angio Biocore, IU Melvin and Bren Simon Cancer center and cultured in SFEM serum-free expansion medium (StemCell Technologies) supplemented with stem cell factor (SCF) (100 ng ml^−1^), thrombopoietin (TPO) (100 ng ml^−1^), and FLT3-ligand (FLT3L) (100 ng ml^−1^). For erythroid differentiation, CD34^+^ HSPCs were cultured in SFEM serum-free medium containing SCF (40 ng ml^−1^) and erythropoietin (EPO) (0.5 U ml^−1^) for around 5 days for further experiments. For generation of lentivirus, HEK293T cells were transfected with the lentiviral constructs described below along with packaging vectors using ProFection Mammalian Transfection System (E1200, Promega) according to the manufacturer’s protocol. Supernatants containing viral particles were collected and filtered with 45 µM filter after 48 h of transfection. For lentiviral transduction, CD34^+^ cells were infected with lentivirus after culturing for 24 h in the presence of 8 µg ml^−1^ polybrene (Sigma-Aldrich) and spun at 800×*g* for 90 min at room temperature.

### Cytospin preparation

A total of 1–2 × 10^5^ cells in 200 μls were used for cytospin using the Thermo Scientific Shandon Cytospin 3 cytocentrifuge. The slides were stained with May-Grünwald solution (Sigma-Aldrich) for 5 min, rinsed in deionized water four times for each 30 s, and subsequently stained with Giemsa solution (Sigma-Aldrich) for 15 min. The images were taken using a Leica inverted microscope.

### Constructs

pRK-Myc-Smurf2(#13678) was provided by Dr Ying Zhang, pCS2-Myc-Cyclin B1(#12176) by Dr Marc Kirschner, and pUHD-HA-CDK1(#27652) by Grag Enders and purchased from Addgene (Cambridge, MA). To generate truncated mutants, C2, WW2, and HECT domain of Smurf2 were cloned by PCR from full-length Smurf2 and inserted into CMV-Myc vectors with the following primers (5′–3′): C2 domain, forward, GAATTCGTCAAGCTGCGCCTGACAGTACTC, reverse, AAGCTTTCAGTCTCTGGACTGAAGACTTACTACTATCTG; WW domain, forward, GAATTCAACGATTTACCAGACGGCTGGGAA, reverse, AAGCTTTCAGTTAGCAGACAGCCGAGGATCTGTAAATTG; HECT domain, forward, GAATTCGCAGGTCATTGCCGCATTGAGGTTTCC, reverse, AAGCTTTCATTCCACAGCAAATCCACATGTTTCTTC. The Flag tagged Ezh2 truncations were generated with the following primers (5′–3′): Ezh2-N (1–348), ATGCGGCGCGCCGGCCAGACTGGGAAGAAATCTG, reverse, TACGTTAATTAATCACTCAGCGGTGAGAGCAGCAGCAAAC; Ezh2-M (330–530), ATGCGGCGCGCCTACCAGCATTTGGAGGGAGCAAAG, reverse, TACGTTAATTAATCAATGATCACAGGGTTGATAGTTGTAAAC; Ezh2-C (523–751), forward, ATGCGGCGCGCCTACAACTATCAACCCTGTGATCATC, reverse, TACGTTAATTAATCAAGGGATTTCCATTTCTCTTTCG.

For knockdown experiments, shRNA sequences targeting human JunD (5′-CTGGAGGATTTACACAAGCAGAACCAGCT-3′), JNK1(5′-GACTCAGAACACAACAAACTT-3′), Bim (5′-GACCGAGAAGGTAGACAATT-3′), Ezh2 (5′-TATGATGGTTAACGGTGATCA-3′), Smurf2 (5′-GATGAGAACACTCCAATTA-3′), and CDK1 (5′-GGGGATTCAGAAATTGATC-3′) were cloned into pCL2 GFP lentiviral vectors provided by Dr. Helmut Hanenberg. The shRNA sequence against mouse Map3k4 (5′-TTACGTCATCTGGACTAAT-3′) was cloned into pMKO.1 GFP retroviral vector which was kindly provided by Dr. William Hahn and purchased from Addgene (#10676).

Double, triple, and quadruple mutations of Ter350, Ter372, Ter419, and Ter492 within Ezh2-M domain into alanine were generated using Quikchange II site directed mutagenesis kit (Agilent) with the following primers (5′–3′): T350A, forward, GAGCGGATAAAGGCCCCACCAAAAC, reverse, GTTTTGGTGGGGCCTTTATCCGCTC; T372A, forward, AGCAGGCCCAGCGCCCCCACCATTA, reverse, TAATGGTGGGGGCGCTGGGCCTGCT; T419A, forward, TCTCGGTGTCAAGCACCAATAAAGA, reverse, TCTTTATTGGTGCTTGACACCGAGA; T492A, forward, GAGGATGTGGATGCTCCTCCAAGGA, reverse, TCCTTGGAGGAGCATCCACATCCTC. The mutations were confirmed by sequencing. Wild type full length Ezh2 and quadruple 4A mutant Ezh2 were cloned into puc2CL6 GFP lentiviral vectors provided by Dr. Helmut Hanenberg.

### Flow cytometry analysis

For analyzing mice erythroblasts, BM cells from P38α^+/−^ and P38α^−/−^ mice were collected and washed with staining buffer (PBS + 5% FBS). Then cells were subsequently stained for 30 min on ice with the following anti-mouse antibodies at a 1:50 dilution:CD71-FITC (553266), Ter119-APC (557909) or Ter119-PE (553673) from BD Biosciences. To evaluate human erythroid differentiation, collected and washed cells were incubated for 30 min on ice with the following anti-human antibodies at a 1:50 dilution: CD235a-PE (12-9987-82, eBioscience), CD71-PerCP/Cy5.5 (334114,Biolegend) or CD71-APC (334108, Biolegend). After wash with staining buffer, the cell pellets were resuspended in staining buffer supplemented with DAPI (Thermo Fisher Scientific) or Propidium iodide (PI) (Sigma-Aldrich). All samples were measured with a BD LSR-II or FACS Calibur (BD Biosciences) and analyzed with Flowjo (Treestar, Ashland, OR, USA).

### Apoptosis and cell cycle analysis

Treated human erythroblasts or mice subset erythroblasts were stained with indicated antibodies against surface markers as described above. To evaluate apoptosis, cells were incubated with APC-conjugated Annexin V (550475, BD Biosciences, 1:50) or PE-conjugated Annexin V (556422, BD Biosciences, 1:50) for 10 min at room temperature in the dark. Then, DAPI or Propidium iodide (PI) was added according to the instructions of the manufacturers. The samples were measured on a BD LSR-II (BD Biosciences) and analyzed with Flowjo.

For cell cycle analysis, human erythroblasts or mice erythroblast subsets were fixed and permeabilized using the BD Cytofix/Cytoperm Fixation/Permeabilization Solution Kit (BD Biosciences) according to the manufacturer’s protocol. Cells were then stained with Ki67 antibody (556027, BD Biosciences, 1:20). DAPI was then added before analysis on a BD LSR-II.

### Fluorescence-activated cell sorting

Briefly, bone marrow cell suspensions from *P38α*^*+/*−^ and *P38α*^−*/*−^ mice were pre-treated with rat anti-mouse CD16/CD32 antibody (2.4G2, BD Biosciences), and subsequently stained with APC-conjugated anti-mouse Ter119 (BD Biosciences,1:50) and FITC-conjugated anti-mouse CD71 (BD Biosciences,1:50) for 30 min in the dark at 4 °C. After wash, cells were stained with Propidium iodide before sorted on BD SORP Aria (BD Biosciences).

### Gene expression profiling analysis and bioinformatics analysis

*P38α*^*+/*−^ and *P38α*^−*/*−^ mice were challenged with PHZ to induce anemia, on day 4 of recovery, erythroblasts (CD71^high^Ter119^+^) from three mice of each genotype were sorted and sent to Miltenyi Biotec Inc (Auburn, CA). RNA was extracted, and gene expression profiling was analyzed using Agilent Whole-Mouse Genome Oligo Microarrays. Data preprocessing and discriminatory gene analysis was conducted by Miltenyi Biotec Inc (Auburn, CA). Based on the log2-tramsformed normalized intensity values, unpaired Student’s *t*-tests (two-tailed, equal variance) was applied to evaluate differences between the means of *P38α*^*+/*−^ and *P38α*^−*/*−^ samples. A correction for multiple testing of the *t*-test *p*-values was conducted using the method of Benjamini and Hochberg. A *p*-value ≤0.05 was used as cutoff. The statistical tests were complemented by a none-statistical quantification of the median expression difference between the two groups. An effect size of 2 (i.e., a fold change of ±2×) was chosen for the selection of candidate reporters.

The identified differentially expressed (DE) genes (fold change >1.5) were used to conduct pathway analyses by Qiagen Ingenuity Pathway Analysis (IPA) (Ingenuity Systems). To perform Gene set enrichment analysis (GSEA), pre-ranked gene list was analyzed by GSEA software (Broad Institute of MIT and Harvard) using the MSigDB gene sets.

### Cell viability assays

Briefly, TF1 cells grown in 96-well plates were treated as described. Then, 10 µL of MTT (5 mg ml^−1^) was added to each well. The plate was incubated at 37 °C for 2 h. Then, DMSO was added to each well and mix thoroughly with the pipette to dissolve the formazan. Absorbance of each sample was read at 570 nm using a microplate reader (Molecular Devices). After substrate of background control, cell viability was expressed as a ratio of absorbance relative to that of control.

### Immunoblotting

Collected cells were lysed in a lysis buffer (20 mM Tris (pH 7.5), 150 mM NaCl, 1 mM glycerolphosphate, 1 mM EDTA, 1 mM EGTA, 2.5 mM sodium pyrophosphate, 1% Triton X-100, 1 mM Na_3_VO_4_, 1 µg ml^−1^ leupeptin (Cell Signaling Technology). The supernatant were obtained by centrifugation at 10,000×*g* for 30 min at 4 °C. Protein concentration was quantified by Bicinchoninic Acid (BCA) Protein Assay kit (Thermo Fisher Scientific). Cell lysates were heated to 95–100 °C for 5 min in sample buffer (187.5 mM Tris-HCl, 6% w/v SDS, 30% glycerol, 150 mM DTT, 0.03% w/v bromphenol blue), and an equal amount of protein was loaded on precast SDS-PAGE (Thermo Fisher Scientific) in running buffer and transferred to nitrocellulose membrane. The membrane was incubated in blocking buffer (1X TBS/T with 5% w/v non-fat dry milk, and then incubated with indicated antibodies according to the manufacturer’s instructions. The Supersignal West Dura extended duration detection system (Thermo Fisher Scientific) was used to expose the membrane to film. Antibodies against P38α (9212,1:1000), phospho-P38 (9211, 1:1000), JNK1(3708, 1:1000), Total JNK (9252, 1:1000), phosphor-JNK (9251,1:1000), Ezh2 (5246, 1:3000), LRF (13097, 1:1000), CDK1(28439, 1:1000), phosphor-CDK1(9111, 1:1000), P53 (2524, 1:1000), Myc tag (2272, 1:1000), and phosphor-T-P (9391, 1:1000) are from Cell Signaling. Anti-P53 (sc-162, 1:1000), anti-Bim (sc-11425, 1:1000), anti-HA (sc-805, 1:1000) are from Santa Cruz Biotechnology. Anti-Flag (T-7425, 1:2000) and anti-actin (A5316, 1:5000) are from Sigma-Aldrich. Anti-JunD (07-1334, 1:1000) and anti-H3K27me3 (07-449, 1:1000) is from Millipore. Original immunoblots are provided in Supplementary Fig 9.

### Retroviral production and transduction

Retroviral pMKO.1GFP vector containing shRNA sequence against mouse Map3k4 was transfected into the Phoenix packaging cell line using ProFection Mammalian Transfection System (E1200, Promega). Supernatants were collected at 48 h post transfection and filtered with 0.45 µM filters. Freshly isolated BM cells were incubated in IMDM containing 20% FBS, 1% penicillin/streptomycin and pre-stimulated with 100 ng ml^−1^ SCF and 10 ng ml^−1^ IL-3 for 48 h prior to retroviral infection on Retronectin (Takara). After transduction for 48 h, GFP positive cells were sorted for further experiments.

### Quantitative real-time PCR

For gene expression analysis, total RNA was extracted using RNeasy Micro Kit (QIAGEN, MD) according to the manufacturer’s protocol. cDNA was synthesized using SuperScript III reverse transcriptase (Thermo Fisher Scientific). Real-time PCR analysis was performed using the SYBR Green Master Mix (Sigma-Aldrich) following the manufacturer’s protocol on a 7500 Real-Time PCR system (Applied Biosystem). The relative expression of target genes was calculated using the delta–delta Ct method and normalized to the β-actin mRNA content. The Primer sequences are showed in Supplementary Tables 1 and 2.

### Immunoprecipitation and co-immunoprecipitation

Cultured HEK293T cells were transfected or co-transfected with indicated plasmids for 48 h. After wash with cold PBS, ice-cold cell lysis buffer (20 mM Tris pH 7.4, 150 mM NaCl, 1 mM EDTA, 1 mM EGTA, 1% Triton X-100, 2.5 mM sodium pyrophosphate, 1 mM β-glycerolphosphate, 1 mM Na_3_VO_4_, protease inhibitor mixture (Sigma-Aldrich) and PMSF) were added to the cells for 30 min. After centrifuge, the supernatants were collected. Pre-cleared lysates were incubated with anti-Flag beads (Sigma-Aldrich) with gentle rocking overnight at 4 °C. Beads were washed three times with cell lysis buffer and suspended in SDS protein loading buffer. The samples were heated to 95–100 °C for 5 min. Immunoprecipitation lysates were analyzed by western blotting with indicated antibodies.

### Chromatin immunoprecipitation (ChIP) assays

After treated with or without Sp600125 (20 µM), human erythroblasts were subjected to the ChIP assay using the EZ-ChIP kit (EMD Millipore) according to the manufacturer’s instructions. Briefly, the cells were cross-linked for 10 min by addition of 37% formaldehyde to a final concentration of 1% at room temperature. 20X glycine were added to quench cross-link. After wash, cell pellets were suspended in SDS Lysis Buffer and sonicated to shear DNA. Collected supernatants were incubated with antibody for overnight at 4 °C with rotation. After elution of protein/DNA complexes and reversal of cross-link, the DNA was purified using spin columns and further analyzed by quantitative PCR. The Primer sequences are showed in supplementary table 3. The antibodies used for CHIP experiments are anti-H3K27me3 (07-449, Millipore,1:500), anti-Ezh2 (5246, Cell Signaling, 1:100).

### Statistical analysis

The animal sample sizes were estimated according to previous studies performed in the similar experiments and the known variability of the assays. The data distribution generally met the assumptions of the tests. All quantitative data are presented as mean ± s.e.m. Two-tailed, unpaired Student’s *t*-tests were performed. Result was considered significant if the *P*-value was below 0.05.

## Electronic supplementary material

Supplementary Information

## Data Availability

Gene expression data have been deposited in the Gene Expression Omnibus (GEO) database under the accession code GSE111751. The authors declare that all the other data supporting the findings of this study are available within the article and its Supplementary Information, or from the corresponding author upon reasonable request.
