# Supplementary material for: P38α/JNK signaling restrains erythropoiesis by suppressing Ezh2-mediated epigenetic silencing of Bim
Source: Nat Commun. 2018 Aug 29;9:3518. doi: 10.1038/s41467-018-05955-2 (PMC6115418; doi:10.1038/s41467-018-05955-2)
Supplement: Supplementary file 1 — Supplementary Information [file 41467_2018_5955_MOESM1_ESM.pdf]

Supplementary Information

P38α/JNK signalling restrains erythropoiesis by suppressing Ezh2-mediated epigenetic silencing of Bim

Hu et al.

Supplementary Figure 1.

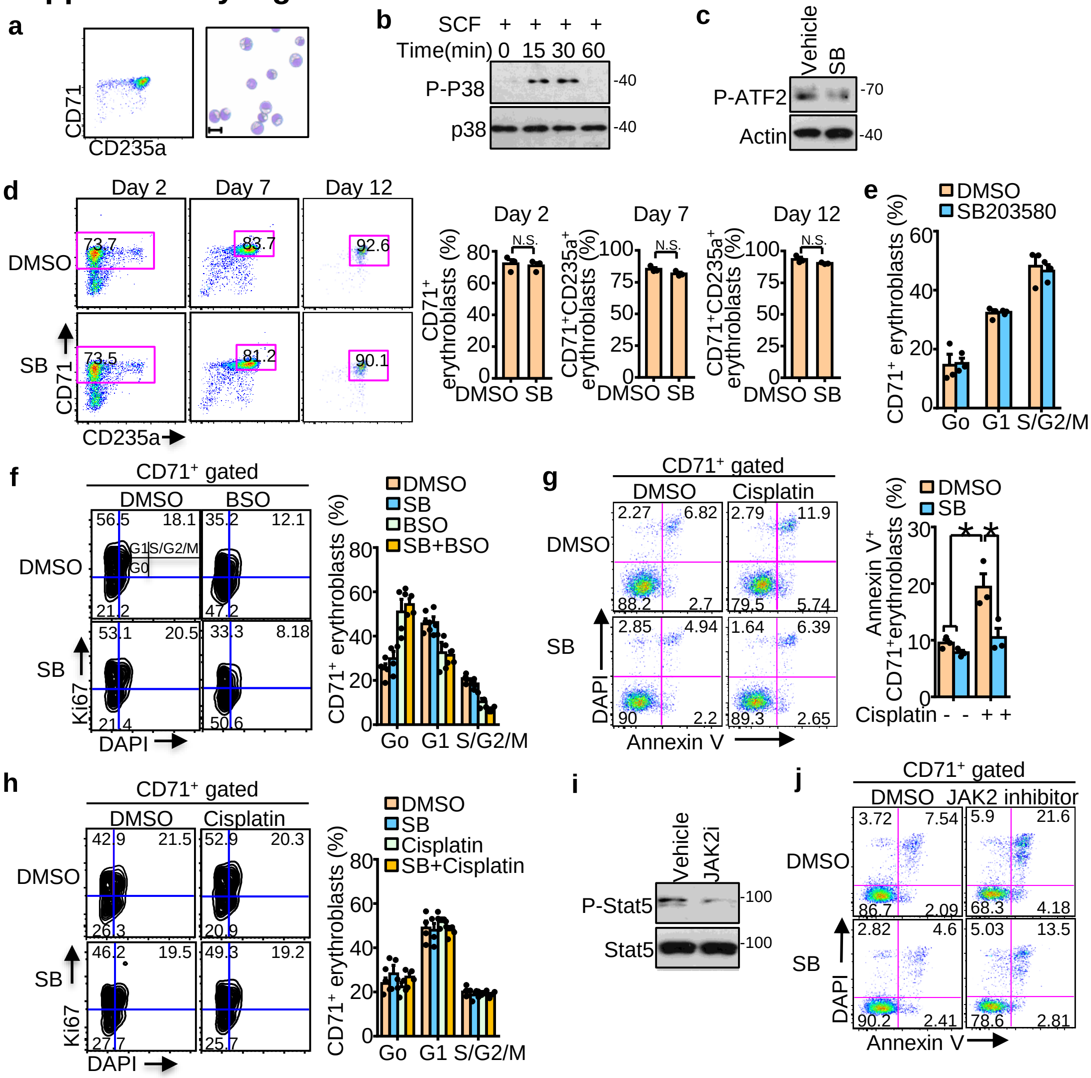

Supplementary Figure 1. Role of P38 in regulation of human erythroblasts.
(a) Representative flow cytometry plot of CD71 and CD235a, showing phenotypic surface marker expression (left) and representative cytocentrifuge image of May-Grünwald-Giemsa staining (right) of human CD34$^+$ HSPCs cells undergoing erythroid differentiation around day 5. Scale bar=20 μM. (b) Phosphorylation of P38 in starved human erythroblasts stimulated with stem cell factor (100 ng ml^{-1}) at indicated time points. (c) Human erythroblasts were cultured with or without SB203580 and phosphorylation of ATF2 was measured by immunoblot. (d) Human CD34$^+$ HSPCs were induced to differentiate into erythroid cells with DMSO or SB203580 (10 μM) and percentage of erythroblasts were determined by flow cytometry with CD71 and CD235a antibody staining. Representative flow cytometry plot (left) and quantification of CD71$^+$ and CD71$^+$CD235a$^+$ cells (right) at the indicated days of differentiation (n=3). (e) Human erythroblasts were treated with DMSO or P38 inhibitor SB203580 (10 μM) and cell cycle stages were analyzed by flow cytometry with Ki67 and DAPI labeling (n=3). (f) Human erythroblasts were incubated with BSO (200 nM) in the presence of DMSO or SB203580 and cell cycle stages of CD71$^+$ cells were measured by flow cytometry as in panel e (n=4). (g,h) Human erythroblasts were incubated with cisplatin (5 μg ml^{-1}) in the presence of DMSO or SB203580. Representative flow cytometry profile (left) and quantification (right) of apoptotic cells (g) and cell cycle distribution (h). (i) Human erythroblasts were cultured with vehicle or Jak2 inhibitor II and phosphorylation of Stat5 was measured by immunoblot. (j) Representative flow cytometry plot showing apoptotic human erythroblasts triggered by Jak2 inhibitor II with DMSO or SB203580. Blots are representative of two independent experiments. Data are shown as mean ± s.e.m. *P < 0.05 (two-tailed unpaired Student's *t*-test)

Supplementary Figure 2.

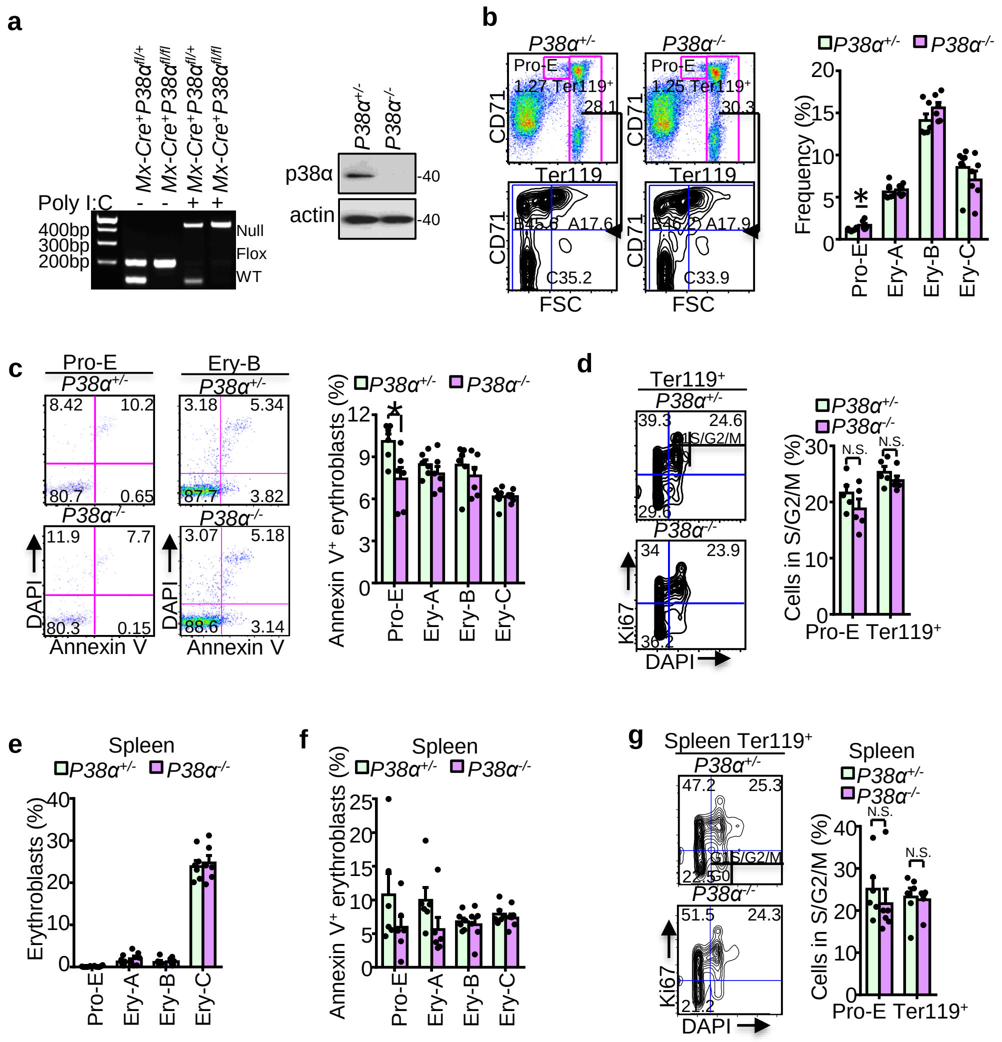

Supplementary Figure 2. Erythroid phenotypes in *P38α*$^{-/-}$ mice.

(a) Excision of *P38α* alleles and expression of P38α protein in erythroblasts from *Mx-Cre*$^{+}$*P38α*$^{fl/+}$ and *Mx-Cre*$^{+}$*P38α*$^{fl/fl}$ mice after polyIC administration were measured by genomic PCR and immunoblotting, respectively. (b-d) Bone marrow erythroblast subsets in *P38α*$^{+/-}$ and *P38α*$^{-/-}$ mice, frequency (n=6) (b), apoptosis (n=6) (c), percentages of Pro-E and Ter119^{+} erythroblasts in S/G2/M phase (n=5) (d). (e-g) Spleen erythroblast subsets in *P38α*$^{+/-}$ and *P38α*$^{-/-}$ mice, frequency (n=6) (e), percentage of apoptosis (f) (n=6) and percentages of Pro-E and Ter119^{+} erythroblasts in S/G2/M phase (n=6) (g). Blots are representative of three independent experiments. Data are shown as mean ± s.e.m. *P < 0.05 (two-tailed unpaired Student's *t*-test)

Supplementary Figure 3.

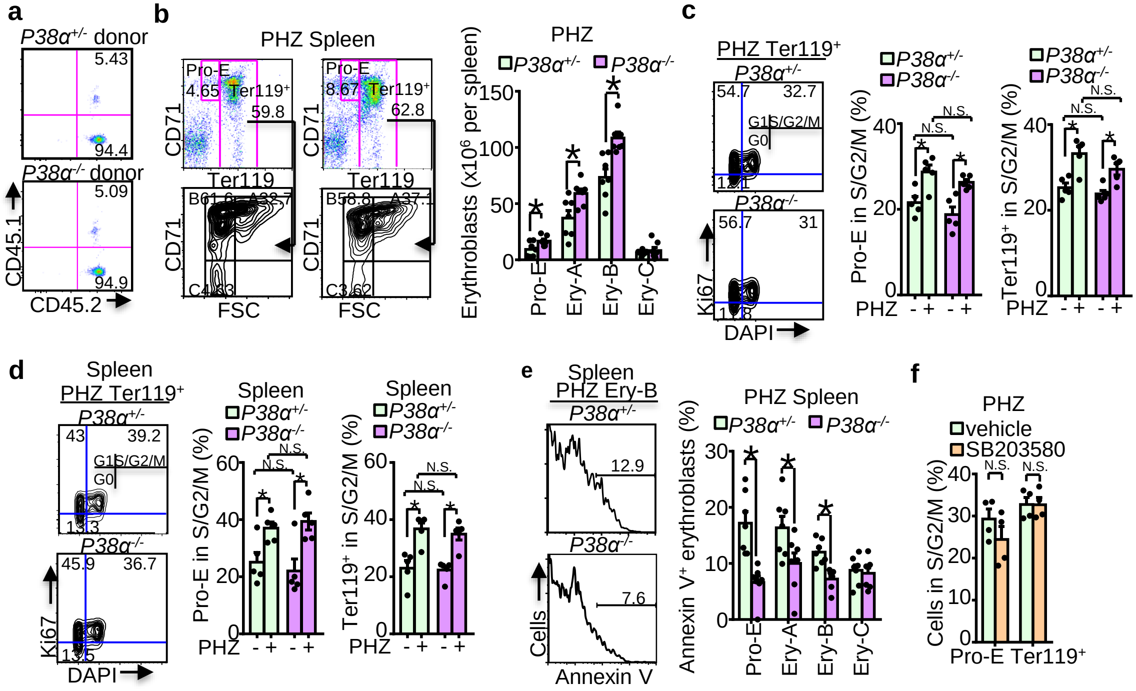

Supplementary Figure 3: Effects of P38α on PHZ-induced stress erythropoiesis. (a) Lethally irradiated C57BL/6-CD45.1 mice were transplanted with bone marrow cells from *P38α*$^{+/-}$ and *P38α*$^{-/-}$ mice. Representative flow cytometry profiles showing percentage of donor-derived cells in PB. (b) Representative flow cytometry profile (left) and numbers of spleen erythroblast subsets in *P38α*$^{+/-}$ and *P38α*$^{-/-}$ mice during recovery (day 4) from PHZ-induced anemia (n=7). (c,d) Percentages of Pro-E and Ter119^{+} erythroblasts in S/G2/M phase in BM (n=5) (c) and Spleen (n=5) (d) in *P38α*$^{+/-}$ and *P38α*$^{-/-}$ mice during anemia recovery from PHZ challenge. (e) Apoptosis of spleen erythroblast subsets in *P38α*$^{+/-}$ and *P38α*$^{-/-}$ mice during recovery from PHZ-induced anemia (n=7). (f) Percentages of BM Pro-E and Ter119^{+} erythroblasts in S/G2/M phase in mice after PHZ challenge treated with vehicle or SB203580 (15mg per kg body weight) (n=4) Data are shown as mean ± s.e.m. *P < 0.05 (two-tailed unpaired Student's *t*-test)

Supplementary Figure 4.

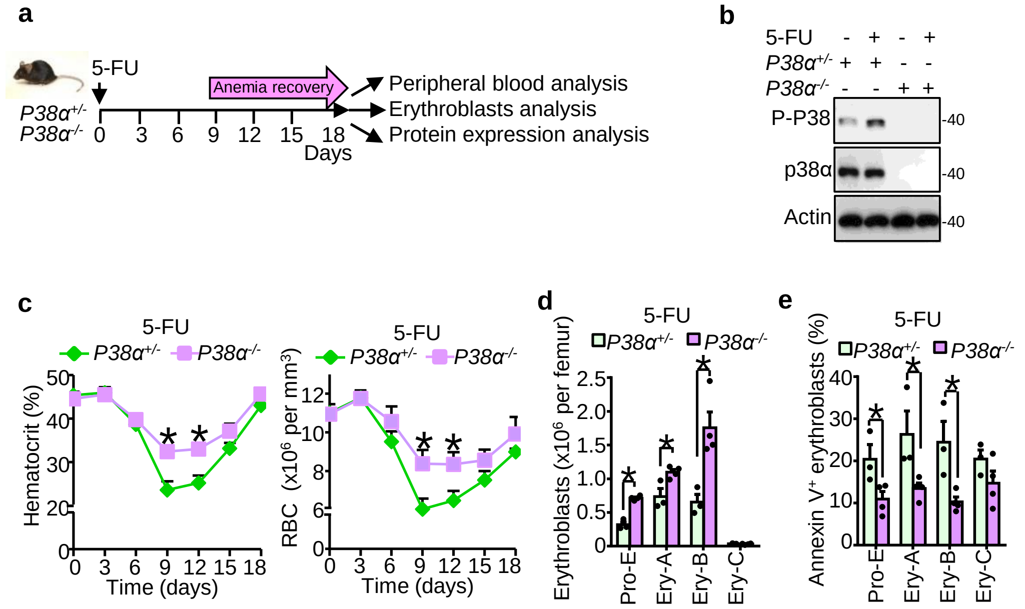

Supplementary Figure 4. Effects of P38α on 5-FU-induced stress erythropoiesis. (**a**) Schematic of 5-FU-induced anemia mouse model. (**b**) During recovery from 5-fluorouracil (5-FU) challenge, phosphorylation of P38 was measured at day 12. (**c**) HCT (left) and RBC counts (right) in *P38α*$^{+/-}$ and *P38α*$^{-/-}$ mice on day 0 (n=10) and indicated times (n=7) during 5-FU-induced anemia. (**d,e**) Numbers of erythroblast subsets (**d**) and apoptosis of erythroblast subsets (**e**) at day 12 after 5-FU challenge in *P38α*$^{+/-}$ (n=3) and *P38α*$^{-/-}$ *mice* (n=4). Blots are representative of three independent experiments. Data are shown as mean ± s.e.m. *P < 0.05 (two-tailed unpaired Student's *t*-test)

Supplementary Figure 5.

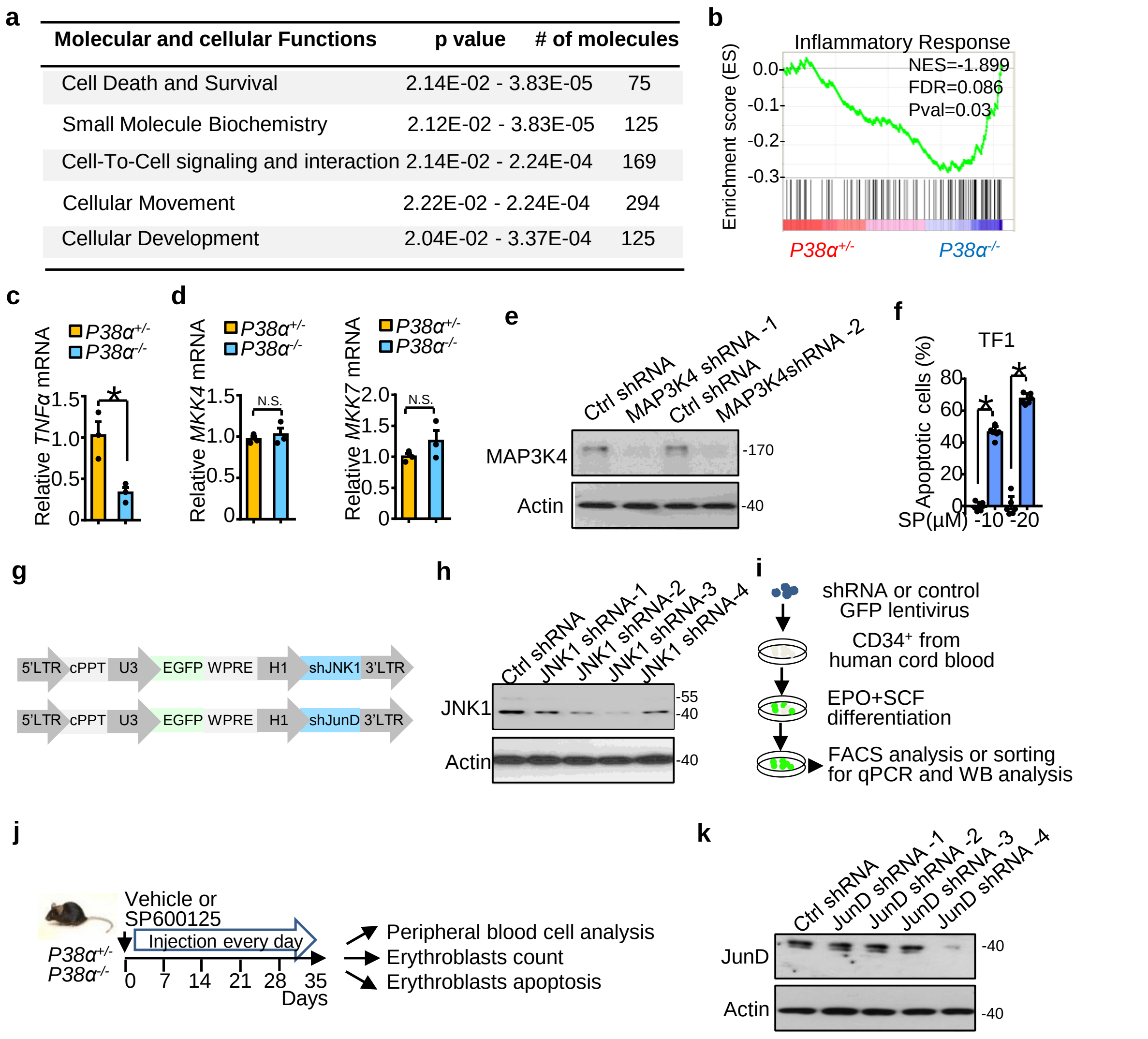

Molecular and cellular Functions	p value	# of molecules
Cell Death and Survival	2.14E-02 - 3.83E-05	75
Small Molecule Biochemistry	2.12E-02 - 3.83E-05	125
Cell-To-Cell signaling and interaction	2.14E-02 - 2.24E-04	169
Cellular Movement	2.22E-02 - 2.24E-04	294
Cellular Development	2.04E-02 - 3.37E-04	125

Supplementary Figure 5. Gene expression profiling and protective role of JNK/JunD in erythroblasts.

(a) Ingenuity pathway analysis shows the top five molecular and cellular functions of P38α-regulated genes and the number of molecules/genes implicated in each category. (b) GSEA plot of "inflammatory response" gene set in *P38α*$^{+/-}$ and *P38α*$^{-/-}$ erythroblasts. (c) q-PCR analysis of the expression of *TNF-α* in *P38α*$^{+/-}$ and *P38α*$^{-/-}$ erythroblasts. (d) mRNA level of *Mkk4* and *Mkk7*, up-stream kinases of JNK, from sorted *P38α*$^{+/-}$ and *P38α*$^{-/-}$ erythroblasts. (e) Confirmation of shRNA-mediated knockdown of mouse Map3k4 in NIH3T3 cells. (f) Cell death of TF1 cells treated with SP600125 measured by MTT assay (n=6). (g) Schematic of pCL2 GFP lentiviral shRNA vector. (h) Confirmation of shRNA-mediated knockdown of JNK1 in Hela cells. (i) Schematic of lentivirus infection of human CD34^{+} HSPCs followed by induced erythroid differentiation for further experiments. (j) Schematic of in vivo treatment of SP600125 on *P38α*$^{+/-}$ and *P38α*$^{-/-}$ mice. (k) Confirmation of JunD shRNA-mediated knockdown in Hela cells. Data are shown as mean ± s.e.m. *P < 0.05 (two-tailed unpaired Student's *t*-test)

Supplementary Figure 6.

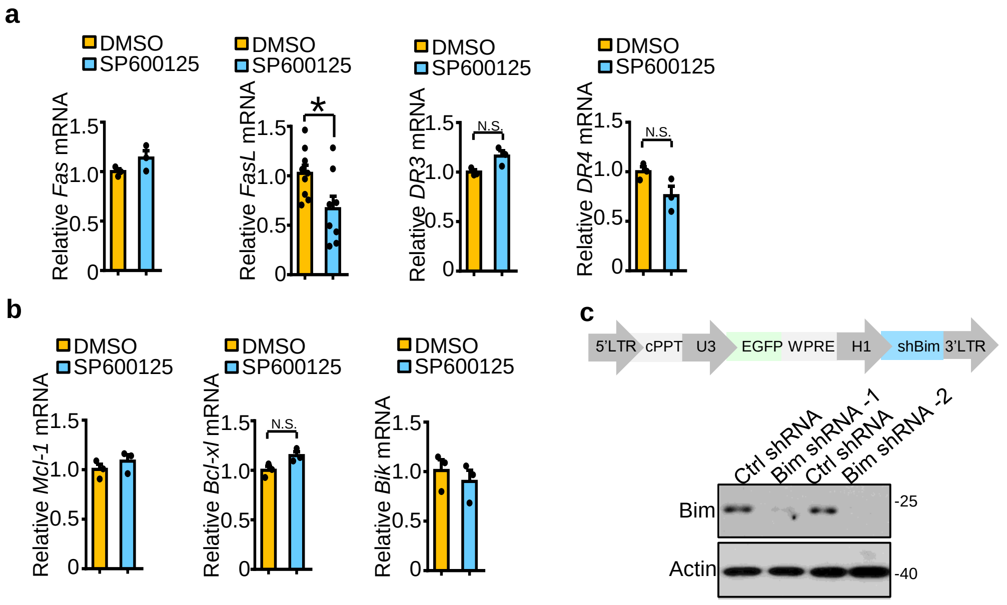

Supplementary Figure 6. Effect of JNK inhibition on expression of genes from death receptor and bcl-2 family.

(a,b) q-PCR analysis on total RNA from human erythroblasts treated with SP600125. Expression of representative genes in the death receptor family (a) and in the bcl-2 family (b). (c) Confirmation of Bim shRNA-mediated knockdown in Hela cells. Data are shown as mean ± s.e.m. *P < 0.05, (two-tailed unpaired Student's *t*-test)

Supplementary Figure 7.

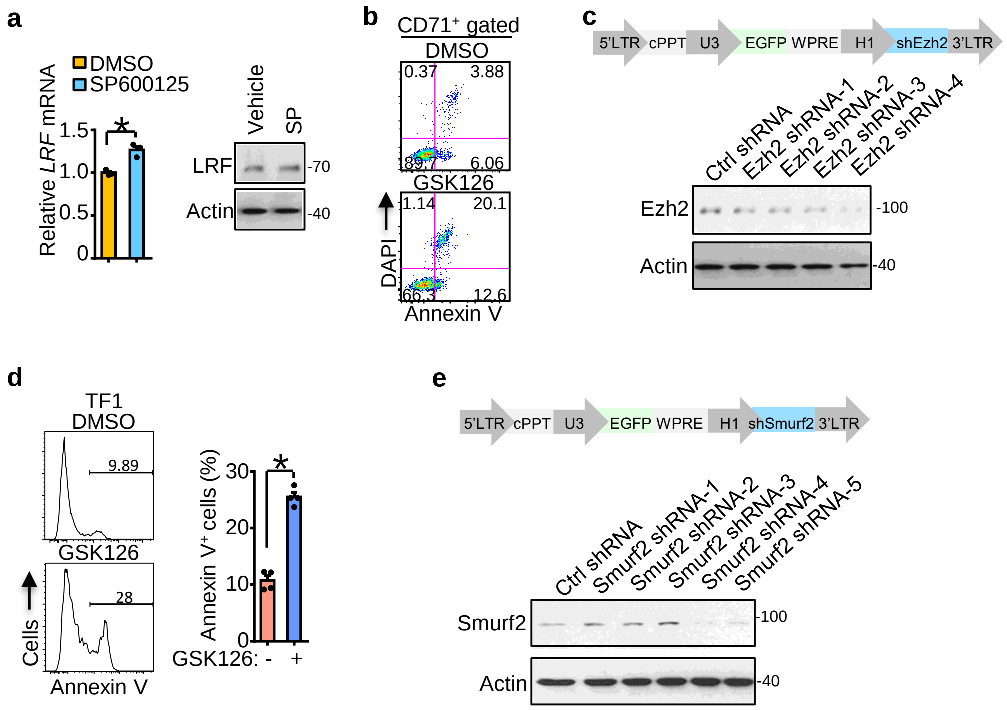

Supplementary Figure 7. Ezh2 but not LRF responses to JNK signaling.
(a) mRNA (left) and protein (right) levels of LRF in SP600125-treated human erythroblasts was analyzed by q-PCR or immunoblotting. (b) Representative flow cytometry plot of apoptotic human erythroblasts induced by GSK126. (c) Confirmation of shRNA-mediated knockdown of Ezh2 in Hela cells. (d) Representative flow cytometry plot (left) and quantification of apoptotic TF1 cells (right) triggered by GSK126 (n=4). Data are mean ± s.e.m. *P < 0.05 (two-tailed unpaired Student's *t*-test) (e) Confirmation of shRNA-mediated knockdown of Smurf2 in Hela cells by immunoblotting.

Supplementary Figure 8.

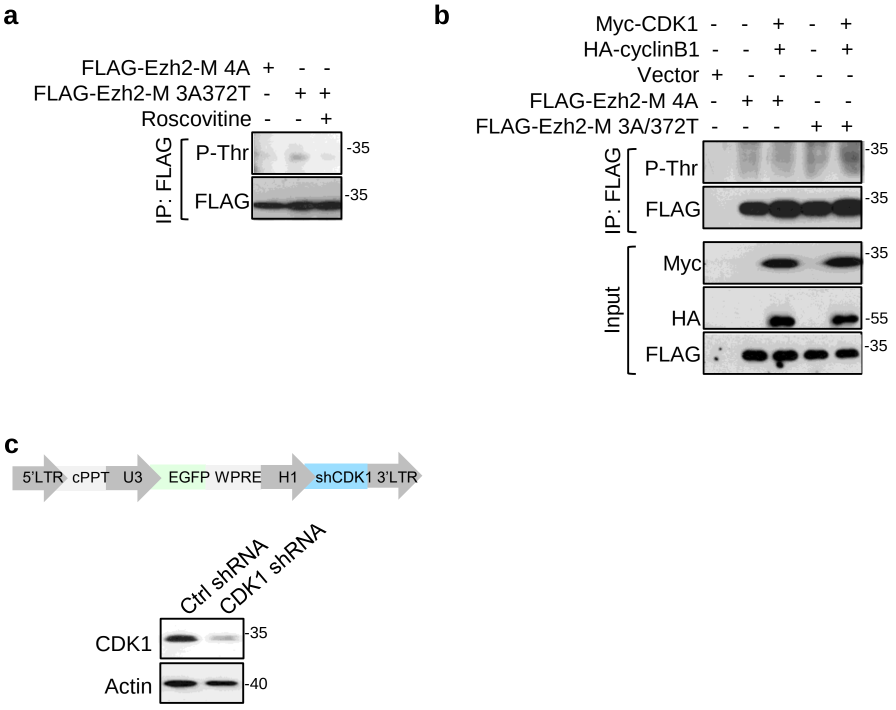

Supplementary Figure 8. T-372 can be phosphorylated by cdk1.

(a) Flag-tagged Ezh2-M 4A and Ezh2-M 3A372T mutants were ectopically expressed in HEK293T cells. After treatment with vehicle or roscovitine (50µM), cell lysates were collected and immuno-precipitated with anti-Flag and immunoblotted with a phosphorylated CDK substrate antibody (pT-P Ab). (b) Flag-tagged Ezh2-M 4A and Ezh2-M 3A372T mutants were co-transfected with Cyclin B and Cdk1 and cell lysates were collected and immuno-precipitated with anti-Flag and immunoblotted with a phosphorylated CDK substrate antibody (pT-P Ab). (c) Confirmation of shRNA-mediated knockdown of Cdk1in Hela cells by immunoblotting.

Supplementary Figure 9.

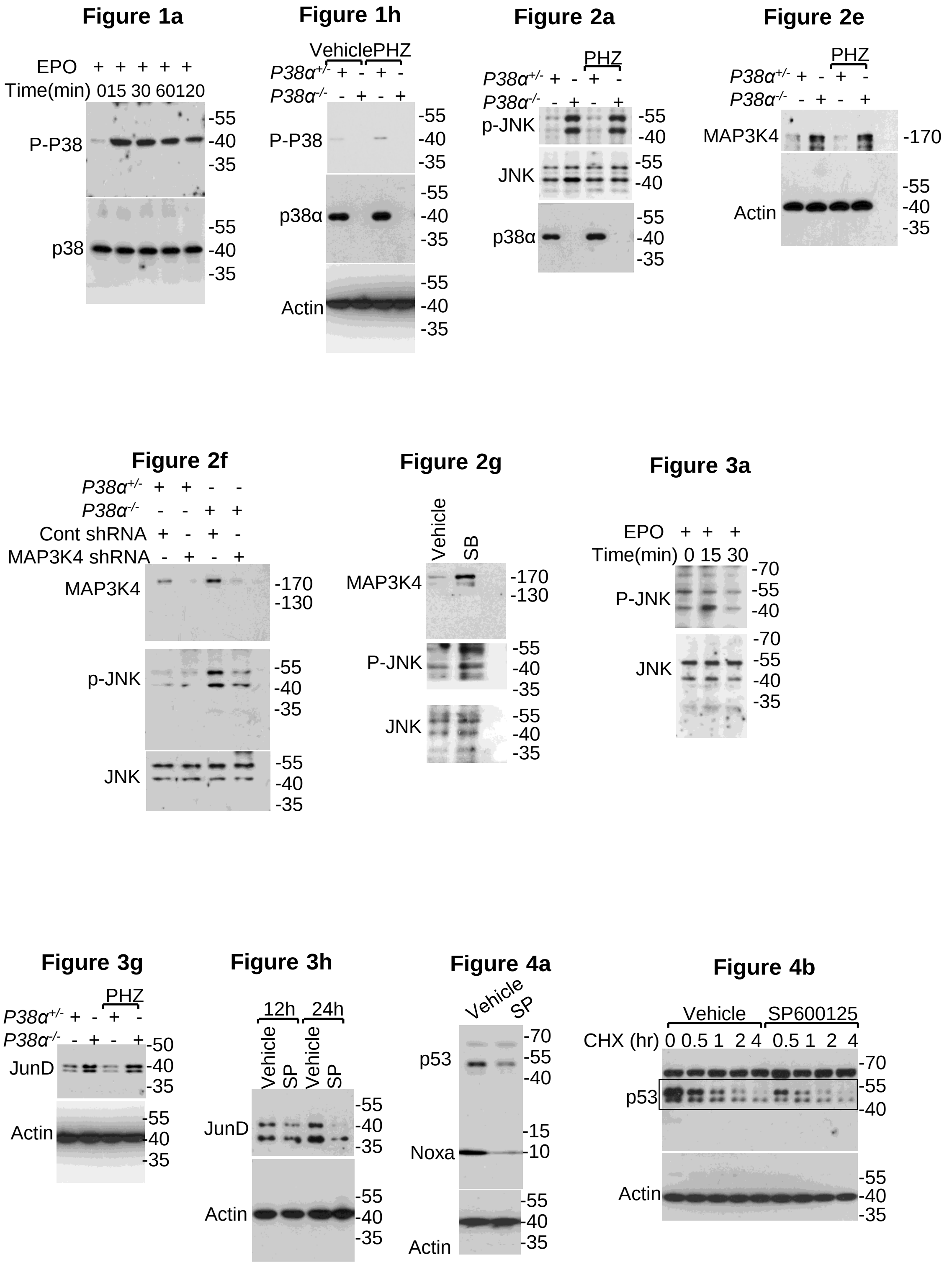

Figure 4c

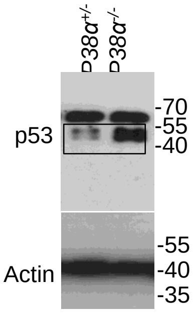

Figure 4e

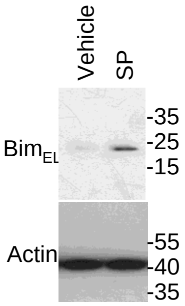

Figure 5a

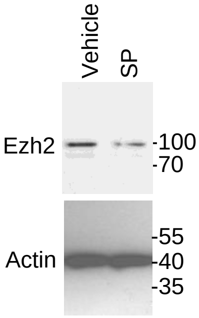

Figure 5b

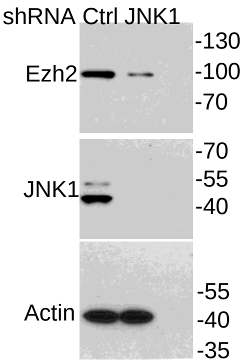

Figure 5c

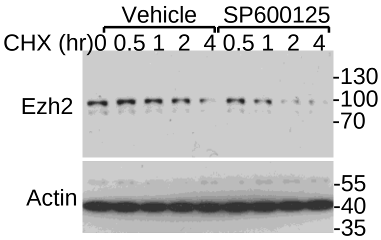

Figure 5d

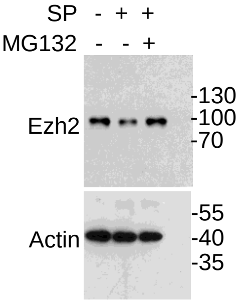

Figure 5g

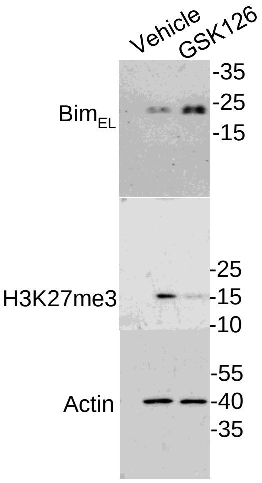

Figure 5k

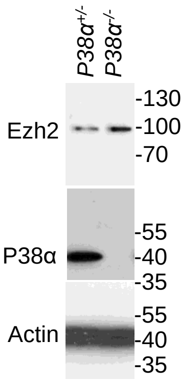

Figure 5n

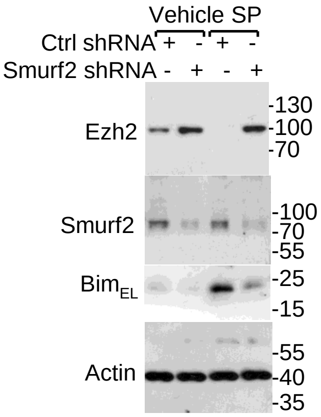

Figure 6a

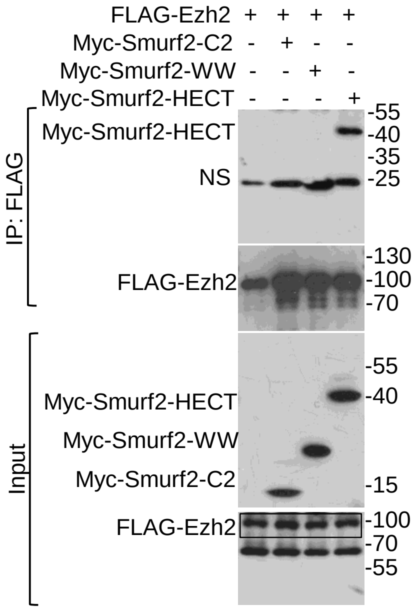

Figure 6b

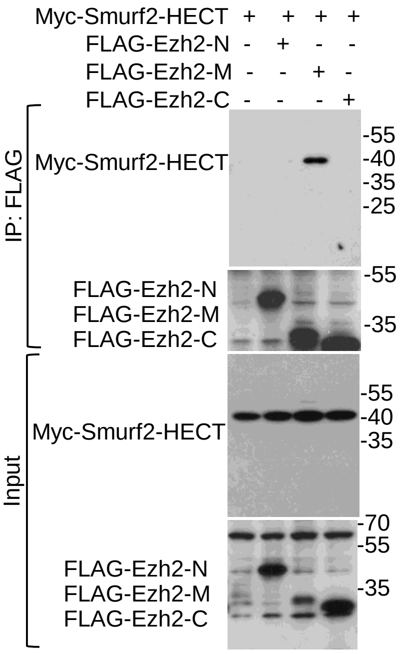

Figure 6c

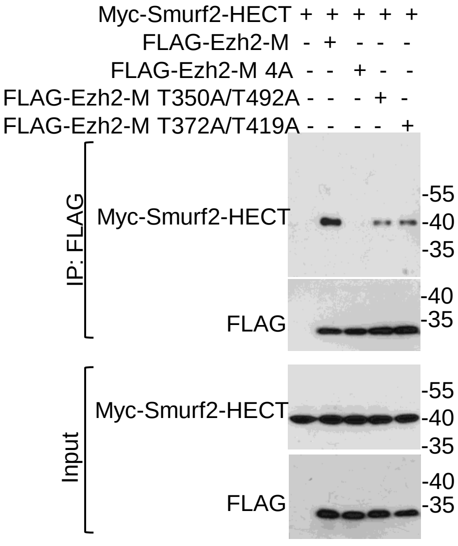

Figure 6d

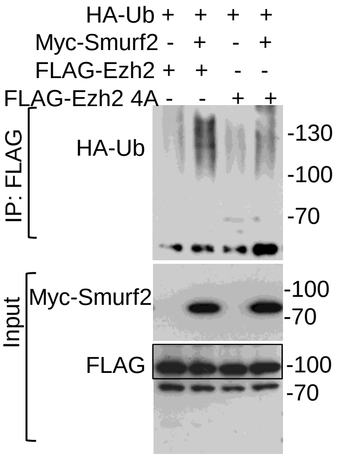

Figure 6e

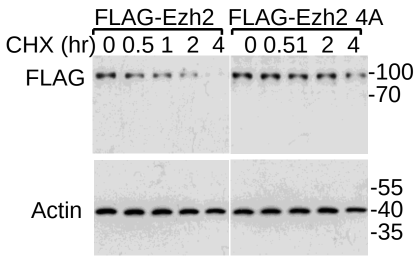

Figure 6f

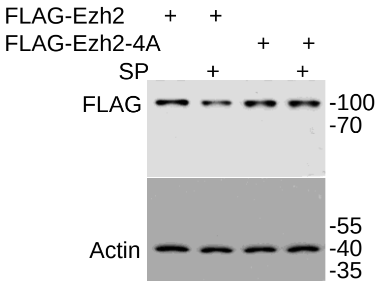

Figure 6g

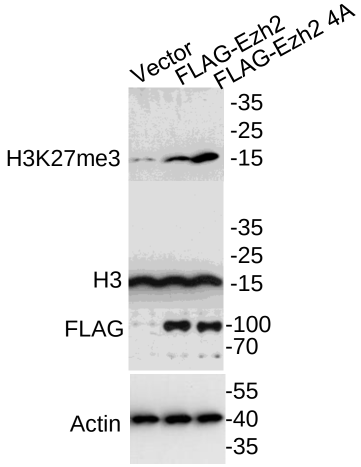

Figure 7a

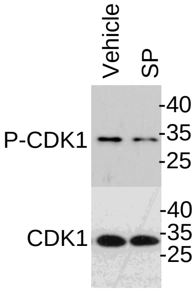

Figure 7b

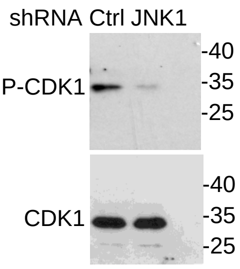

Figure 7c

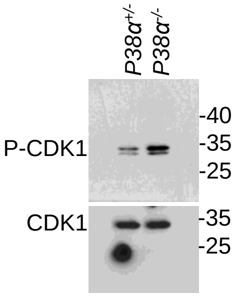

Figure 7d

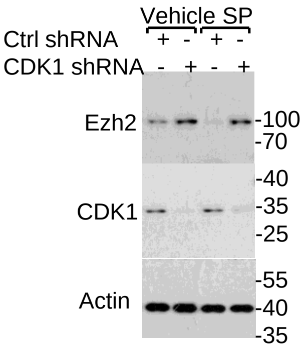

Supplementary Figure 9: Original immunoblots

Supplementary tables

Table 1. Mouse Expression Primers

Targets	Sequence
*MKK3*_F	GAAGCAGGTGGTGGAGGAG
*MKK3*_R	GCAAGGTGAAGAATGGGTGT
*MKK6*_F	GCCATCCTTCGGTTTCCTT
*MKK6*_R	CTGCTGGGAGCTGAGGAGAG
*Map3k4*_F	CTCCTCGACCACGCTTTTGT
*Map3k4*_R	CCTGCGCTGCAGTCTTTATG
*Map3k11*_F	GCCCATAGTTCACCCACTCC
*Map3k11*_R	AGGTGTGCCAGGAGCAGAG
*MKK4*_F	CAAGAGATGCTGGGTGTAGG
*MKK4*_R	GATAAGGAAATCGGCCTGTG
MKK7-F	ATCCGAGCTGATGTGTGGA
MKK7-R	ATGTGACCAGGCAGGAGTG
*GADD45g*_F	GGGAAAGCACTGCACGAACT
*GADD45g*_R	AGCACGCAAAAGGTCACATTG
*JunD*_F	TTGGTTGCGTGTTGAGTGTG
*JunD*_R	ATGGTGTCGGGAGGAGAGAG
*HOXA9*_F	CCCACGCTTGACACTCACACTTTG
*HOXA9*_R	GAGTGGAGCGAGCATGTAG
*TNF-α*_F	GTCCCCAAAGGGATGAGAAG
*TNF-α*_R	CACTTGGTGGTTTGCTACGA
*EPOR*_F	GGGCTCCGAAGAACTTCTGTG
*EPOR*_R	ATGACTTTCGTGACTCACCCT
*TFR2*_F	TTGGGGTCTACTTCGGAGAGT
*TFR2*_R	GACAGGAGCCTAAGTGCTCAG
*P53*_F	GATGACTGCCATGGAGGAGT
*P53*_R	GTCCATGCAGTGAGGTGATG
*Actin*_F	GACGGCCAGGTCATCACTATTG
*Actin*_R	AGGAAGGCTGGAAAAGAGCC

Table 2. Human Expression Primers

Targets	Sequence
*Bim*_F	GATCCTCCAGTGGGTATTTCTCTT
*Bim*_R	ACTGAGATAGTGGTTGAAGGCCTGG
*LRF*_F	TACGAGTGCAACATCTGCAAG
*LRF*_R	AGGTCGTAGTTGTGGGCAAAG
*Ezh2*_F	TTGCCAAGAGAGCCATCCA
*Ezh2*_R	GCATCAGCCTGGCTGTATCTG
*Fas*_F	CCGGACCCAGAATACCAAG
*Fas*_R	GAAGACAAAGCCACCCCAAG
FasL-F	CTACCAGCCAGATGCACACA
FasL-R	CCATAGGTGTCTTCCCATTCC
*DR3*_F	CAGATGAAGCTGGGATGGAG
*DR3*_R	GAGGTGCTAGAAGGGTGTGG
*DR4*_F	GTTGTTCCGTTGCTGTTGGT
*DR4*_R	GCGTTGCTCAGAATCTCGTT
*Mcl-1*_F	GGGCAGGATTGTGACTCTCATT
*Mcl-1*_R	GATGCAGCTTTCTTGGTTTATGG
*Bcl-XL*_F	GAATGACCACCTAGAGCCTTGG
*Bcl-XL*_R	TGTTCCCATAGAGTTCCACAAAAG
*Bik*_F	GAGATGGACGTGAGCCTCAG
*Bik*_R	TGATGTCCTCAGTCTGGTCG
*HOXA9*_F	TACGTGGACTCGTTCCTGCT
*HOXA9*_R	CGTCGCCTTGGACTGGAAG
*Actin*_F	AGAGCTACGAGCTGCCTGAC
*Actin*_R	AGCACTGTGTTGGCGTACAG

Table 3. CHIP Primers

*Bim promoter*_F	GGGAGGCTAGGGTACACTTC
*Bim promoter*_R	TGGCGTGTTTACCGGAGTA
